# Integrative Analysis of Deep Sequencing Data Identifies Estrogen Receptor Early Response Genes and Links *ATAD3B* to Poor Survival in Breast Cancer

**DOI:** 10.1371/journal.pcbi.1003100

**Published:** 2013-06-20

**Authors:** Kristian Ovaska, Filomena Matarese, Korbinian Grote, Iryna Charapitsa, Alejandra Cervera, Chengyu Liu, George Reid, Martin Seifert, Hendrik G. Stunnenberg, Sampsa Hautaniemi

**Affiliations:** 1 Research Programs Unit, Genome-Scale Biology and Institute of Biomedicine, University of Helsinki, Helsinki, Finland; 2 Department of Molecular Biology, Faculty of Science, Nijmegen Center for Molecular Life Sciences, Radboud University, Nijmegen, The Netherlands; 3 Genomatix Software GmbH, Munich, Germany; 4 Institute of Molecular Biology, Mainz, Germany; National University of Singapore, Singapore

## Abstract

Identification of responsive genes to an extra-cellular cue enables characterization of pathophysiologically crucial biological processes. Deep sequencing technologies provide a powerful means to identify responsive genes, which creates a need for computational methods able to analyze dynamic and multi-level deep sequencing data. To answer this need we introduce here a data-driven algorithm, SPINLONG, which is designed to search for genes that match the user-defined hypotheses or models. SPINLONG is applicable to various experimental setups measuring several molecular markers in parallel. To demonstrate the SPINLONG approach, we analyzed ChIP-seq data reporting PolII, estrogen receptor 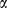 (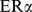), H3K4me3 and H2A.Z occupancy at five time points in the MCF-7 breast cancer cell line after estradiol stimulus. We obtained 777 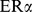 early responsive genes and compared the biological functions of the genes having 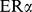 binding within 20 kb of the transcription start site (TSS) to genes without such binding site. Our results show that the non-genomic action of 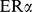 via the MAPK pathway, instead of direct 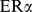 binding, may be responsible for early cell responses to 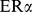 activation. Our results also indicate that the 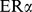 responsive genes triggered by the genomic pathway are transcribed faster than those without 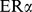 binding sites. The survival analysis of the 777 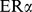 responsive genes with 150 primary breast cancer tumors and in two independent validation cohorts indicated the *ATAD3B* gene, which does not have 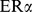 binding site within 20 kb of its TSS, to be significantly associated with poor patient survival.

## Author Summary

Cellular processes in mammalian cells are tightly regulated to ensure that the cells function properly as a part of an organism. Dysregulation of some of these processes, such as apoptosis, cell proliferation and growth, can lead to cancer. One of the most important regulation mechanisms for cellular processes is via activation of membrane receptors by extra-cellular stimulus. Such cues trigger signal cascades that lead to altered expression of a number of genes in the cell nucleus; a key challenge in biomedicine is to identify which genes respond to a specific stimulus. These so called response genes can be investigated on a whole-genome scale with genomic sequencing, which is a technology that can quantify protein binding to DNA or gene activation. Analysis of such whole-genome data, however, is challenging due to billions of data points measured in the experiments. Here we introduce a novel computational method, SPINLONG, which is a widely applicable novel computational method that integrates multiple levels of deep sequencing data to produce experimentally testable hypotheses. We applied SPINLONG to breast cancer data and found early responsive genes for estrogen receptor and analyzed their regulation. These analyses resulted in a gene whose high activity is associated with decreased breast cancer patient survival.

## Introduction

The identification of genes whose expression patterns are altered due to a stimulus is essential as it provides a basis to understand which signaling and metabolic pathways are influenced as a consequence of a stimulus. The majority of approaches to identify stimulus-regulated changes in gene expression rely on the relative abundance of mRNA molecules, either measured with microarrays or with RNA-seq, as an indirect indication of transcriptional initiation [1]–[3]. A major issue with using full length mRNA molecules as an indication of a transcriptional response is that the time needed to transcribe full length mRNA molecules depends strongly on the length of the genes: Whereas the transcription of short genes (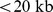) can be completed within less than 10 minutes, longer genes may take over an hour to be transcribed. Consequently, secondary responses, which may occur before the longest genes are fully transcribed, make identification of primary responsive genes challenging.

Transcription is a dynamic process that is regulated by transcription factors and is reflected in local histone modifications. A reliable indication of an actively transcribed gene is the presence of RNA polymerase II (PolII) protein complex in the body of the gene. PolII generates the precursors of most mRNA, snRNA and miRNA molecules, and its activity is modulated by histone modifications [4]. Chromatin-level phenomena predict the majority of RNA level changes [5], and the changes in PolII activity after a stimulus are detectable earlier than changes in mature RNA levels. Thus, we hypothesized that considering PolII together with histone modifications could provide a reliable indication of changes in the rate of transcriptional activity at responding loci.

Genome-wide PolII activity can be measured with ChIP-seq (chromatin immunoprecipitation combined with massive parallel sequencing) [6] and with GRO-seq (global run-on sequencing) [7]. The PolII machinery moves through the body of a transcribed gene, and following stimulation this motion can be seen as a ‘wave’ of increased reads along the responding genes. Conceptually, the PolII wave is a spatio-temporal pattern. Here, we introduce a data-driven computational approach, SPINLONG (Spatial Pattern Identification by Non-Linear OptimizatioN with Global constraints), that identifies spatio-temporal patterns in deep sequencing data. To accommodate for various experimental setups, SPINLONG allows users to define custom searchable patterns. For instance, such spatial patterns could be: ‘low-low’ (little PolII activity without stimulation), ‘high-low’ (increased number of reads at the beginning of a gene, but not at the end) and ‘high-high’ (fully transcribed gene characterized by a large number of reads along the gene). To our knowledge, SPINLONG is the first approach that allows identification of such spatio-temporal patterns from deep sequencing data.

Estrogen receptor 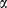 (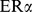) is over-expressed in 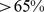 of breast cancers and is a major therapeutic target in breast cancer [8]. Extra-cellular estradiol actives 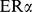, which triggers cytoplasmic signaling cascades as well as transport of the activated 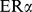 complex to the nucleus where 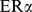 acts as a transcription factor. These lead to changes in expression patterns of hundreds of 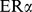 responsive genes that govern cell phenotypes, such as cell growth and proliferation. The importance of 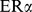-mediated gene regulation in breast cancer was highlighted in a recent study that showed that genes with an elevated expression pattern and an 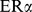 binding site within 20 kb from their transcription starting sites are correlated with patient survival [3].

In order to demonstrate the utility of SPINLONG, we characterized estradiol-induced early responsive genes in MCF-7 breast cancer cells with SPINLONG analysis based upon ChIP-seq data for PolII, H3K4me3 and H2A.Z occupancy at 5+2 time points following estradiol stimulation. Time points 0, 10, 20, 40 and 80 minutes were used to capture early transcriptional responses. Additionally, information from 160 and 640 minutes was used as auxiliary data points to supplement the main analysis. We used also ChIP-seq data for measuring the binding of 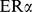 to DNA at 5 minutes time point to identify genes that are directly regulated by 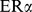 binding. After identifying the 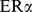 early responsive genes, we used 150 primary breast cancer samples from The Cancer Genome Atlas [9] to assess the survival effect of the 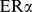 response genes. All results are available at http://csbi.ltdk.helsinki.fi/spinlong/mcf7/ and in Supporting Information. All deep sequencing data are available at http://csblsynergy.fimm.fi/.

## Results

### The SPINLONG method

**Figure 1:**
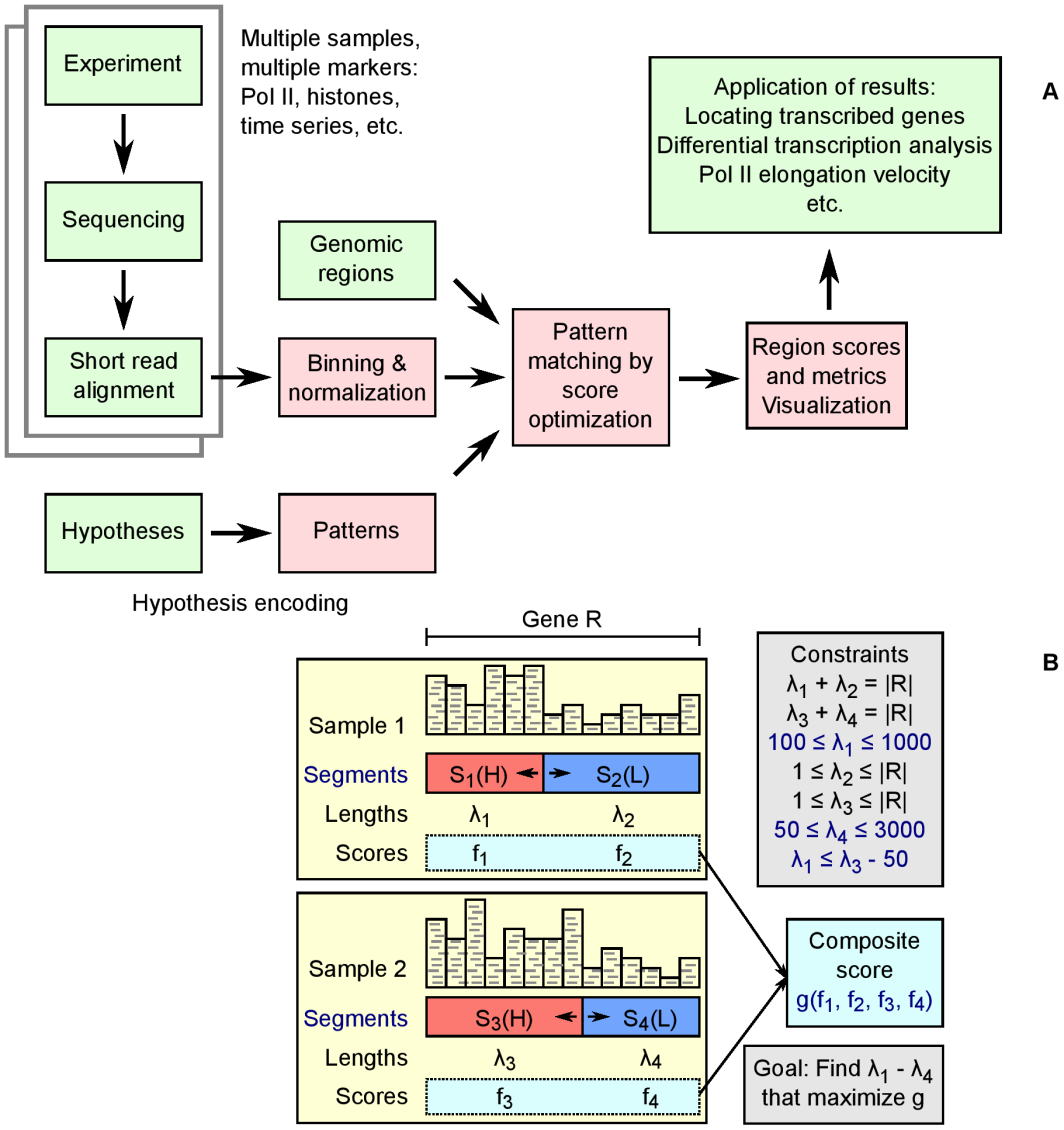
(A) Schematic workflow of SPINLONG. Red and green boxes denote implementation of SPINLONG and input from a user, respectively. SPINLONG produces tabulated and graphical results that can be interpreted in the context of the hypotheses. (B) Illustration of the pattern matching step with two samples and four segments in the context of a gene 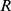. User-provided information is shown with blue labels. Short reads (gray) for each sample are assigned into fixed length bins and bin counts are normalized. A user-defined pattern describes the expected spatial distribution of bin counts in the samples, formulated by dividing each sample into one or more segments (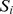). Segment classes *H* and *L* indicate whether the segment is expected to contain a “high” (red) or “low” (blue) amount of short reads, respectively. The user defines the segment divisions and their classes, while nonlinear optimization is used to assign segment lengths (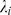) by maximizing score function 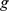 and taking into account linear constraints. The composite score 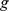 is composed of segment scores 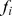, which are computed based on bin counts and segment lengths. Constraints include structural constraints, such as the requirement that segment lengths of a sample sum up to gene length 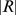, as well as custom constraints (blue).

SPINLONG is a computational method for ranking genomic regions, such as genes, based on how closely they match a spatio-temporal deep sequencing pattern defined by the user. The overall schematic of the SPINLONG approach is shown in Figure 1 and a detailed description is in Materials and Methods. Briefly, the user first encodes a set of hypotheses, or models, as patterns for SPINLONG. These patterns divide each genomic region into one or more contiguous segments that are expected to contain a “low” or “high” number of sequencing reads. Patterns are configured using an expressive notation based on linear algebra, supporting complex experimental setups such as time series and multiple molecular markers. A deep sequencing experiment, such as ChIP-seq, is followed by preprocessing in SPINLONG. In the primary analysis step scores are assigned to each pattern for each defined genomic region utilizing Hidden Markov Models. The scores are computed by a non-linear optimization procedure which assigns lengths to each segment in the patterns and estimates numeric short read thresholds for “low” and “high” segments. The scoring method and parameters can be configured by the user. The resulting scores and accompanying metrics indicate how well the pattern observed in the data matches the anticipated pattern defined by the user. Scores can be used to classify genes as induced or repressed, whereas assigned segment lengths can be used in downstream analyses, such as estimating PolII elongation speed.

### Identifying PolII activity regions in MCF-7 after estradiol stimulus

An essential first step in identifying 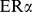 genes with the SPINLONG pattern matching process is to accurately establish genomic regions of PolII activity that correspond to gene promoter and body. These regions correlate with transcribed units delimited by transcription start sites (TSSs) and termination sites (TTSs). While *a priori* coordinates of transcribed regions can be obtained from genomic databases that list annotated genomic features, these do not take context dependent factors into account. For instance, individual transcripts often have multiple TSSs, with specific TSSs utilized in response to particular signaling processes [10], [11]. Consequently, we used SPINLONG to determine regions of PolII activity directly from the deep sequencing data in a data-driven fashion.

In addition to PolII ChIP-seq profiles, local histone modifications and substitution by the variant histone H2A.Z correlate with the location of TSSs and thus provide information that can be used in localizing the gene bodies [4], [12]. We therefore used PolII in conjunction with H3K4me3 and H2A.Z ChIP-seq data to delimit estrogen responsive gene regions in MCF-7. Occupancy of PolII over an annotated transcript is used to locate both PolII activity start and termination sites (corresponding roughly to TSSs and TTSs), while the location of H3K4me3 and H2A.Z correlate with the location of start sites. Initial coordinates of transcribed loci were obtained from Ensembl v.61 [13] and then extended in the 5′ and 3′ directions by 50% of the gene length, to generate a total region for analysis twice the length of the transcribed unit.

Regions associated with the presence of PolII are computed from the lengths of PolII body segments assigned by the SPINLONG score optimizer (see Materials and Methods). The scores ranged from 0 to 1.08, and the threshold used here (0.60) corresponds to a gene whose spatial short read coverage matches the evaluated pattern in 60% of genomic locations. The spatial pattern searched from the PolII profile is ‘low-high-low’, *i.e.*, a gene body with a large number of PolII reads is flanked by areas of low PolII signal. If there are overlapping transcribed genes within the area of search, the resulting spatial distribution matches poorly with this pattern and produces a low score. For such genes and others with a low score, Ensembl regions are used. This criterion, when combined with local H3K4me3 and H2A.Z profiles, identified 4,275 genes that were considered as active PolII regions.

**Figure 2:**
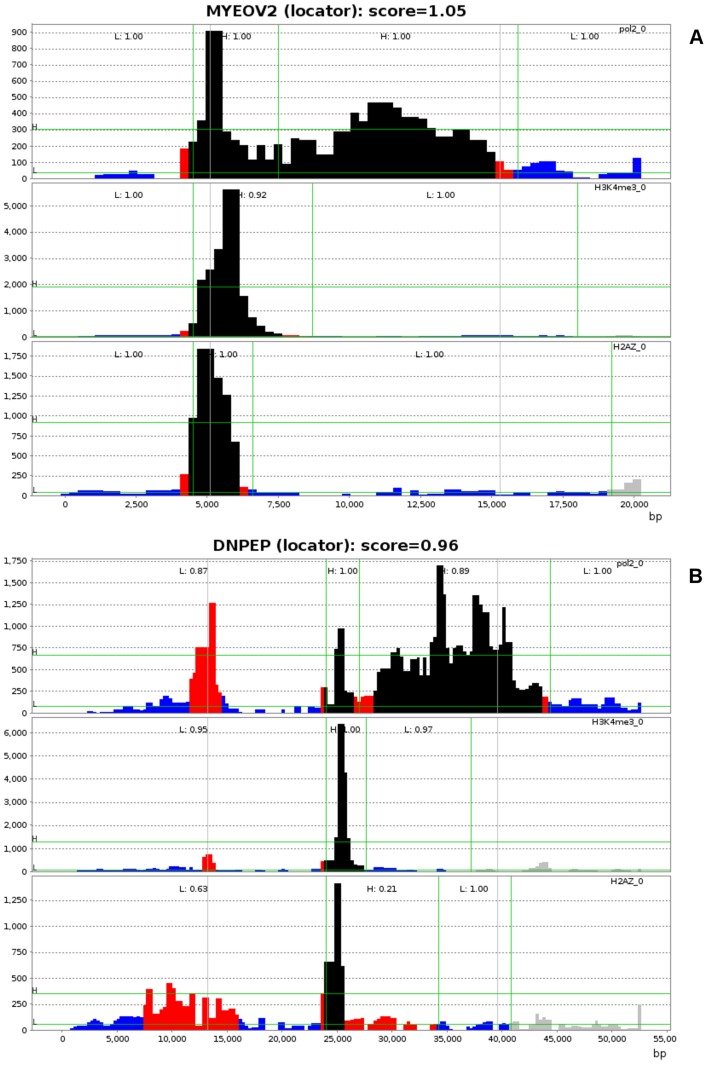
Locating the transcribed region of (A) *MYEOV2* and (B) *DNPEP*. PolII, H3K4me3 and H2A.Z read counts are shown vertically with sample labels on the right. Sample data are the maximum of bin counts over all time points. The X-axis shows gene position in base-pairs (5′ to 3′ direction) and the Y-axis shows relative bin count. Green horizontal lines indicate Gaussian means of HMM states; the HMM state with lower mean is interpreted as class *L* state and the higher as class *H* state. Green vertical lines indicate segment boundaries assigned using simulated annealing. Gray vertical lines indicate gene locations obtained from Ensembl. The class and score of each segment is shown on top. For bin counts, black indicates a bin count inside class *H* segment that contributes positively to segment score and blue is analogous for class *L*; red is a mismatching bin. Transcribed regions are obtained by concatenating the two H class segments of PolII.

Two high scoring putative estrogen responsive genes, *MYEOV2* and *DNPEP*, are shown in Figure 2. *MYEOV2*, which has score of 1.05, has proximal promoter PolII enrichment that colocalizes with H3K4me3 and H2A.Z peaks, and the PolII signal in the body of the *MYEOV2* is well above that of the 5′ and 3′ flanking regions. The extent of occupancy of PolII, H3K4me3 and H2A.Z on *MYEOV2* makes locating the PolII activity region relatively straightforward. In contrast, *DNPEP* (score 0.96) has multiple known potential promoter regions and over 20 alternative transcripts. Taking into account H3K4me3 and H2A.Z occupancy, the SPINLONG optimizer selected a region that initiates 10 kb downstream from the annotated TSS.

### Early responding genes upon estradiol stimulus of MCF-7 cells

**Figure 3:**
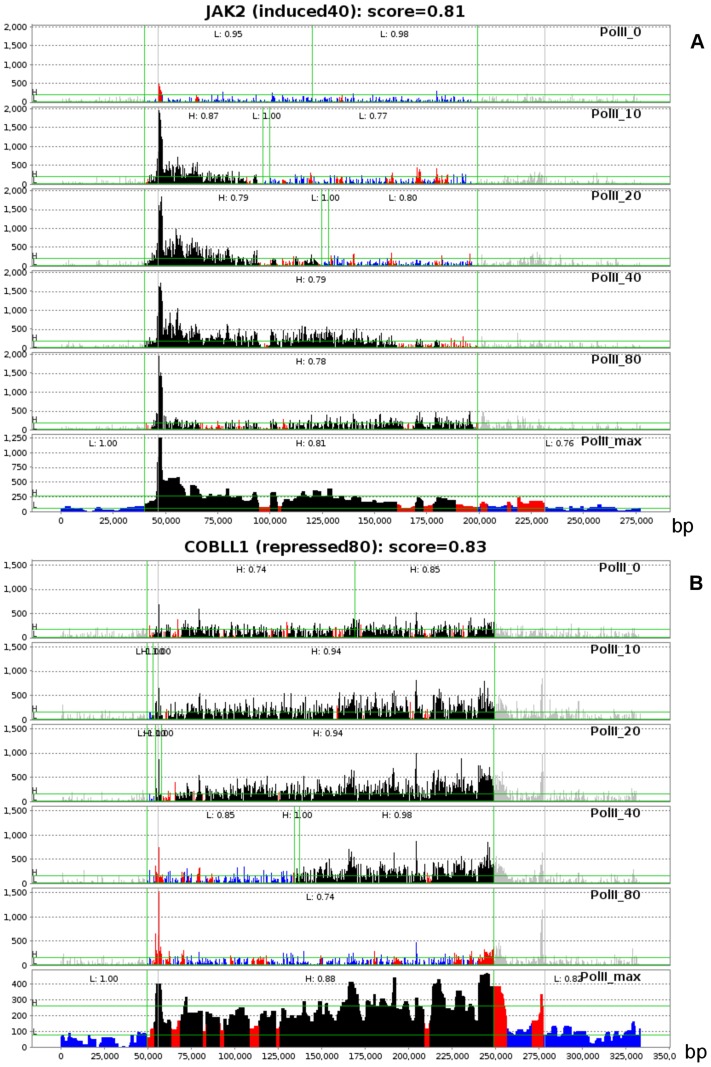
Examples of (A) an estradiol induced gene (*JAK2*) and (B) a repressed gene (*COBLL1*). PolII read counts are shown vertically. The last pseudo-sample (locator) is an aggregate sample containing the maximum value of all time points used for fine-tuning the gene location. Initial locations (gray vertical lines) are obtained from a previous optimizer run with PolII and histone markers. Gray bins indicate they are within ignored area (class *N*). (A) *JAK2* shows a leading edge that progresses gradually and reaches the end of the gene by 40–80 minutes. Transcriptional response starts fading at 80 minutes. (B) *COBLL1* shows a similar lagging edge that reaches the end of the gene by 80 minutes.

Using five time-points of PolII ChIP-seq measurements (0, 10, 20, 40 and 80 minutes after stimulation), in conjunction with a non-specific antibody background sample as a subtraction control and utilizing a conservative score threshold (0.65) for an estrogen regulated gene, SPINLONG analysis characterized 699 genes as induced and 78 as repressed following estradiol stimulus. The results are available at http://csbi.ltdk.helsinki.fi/spinlong/mcf7/. Figure 3 illustrates *JAK2*, which is induced after stimulation of MCF-7 cells with estradiol; and *COBLL1*, which is repressed after the stimulation. Both are relatively long (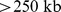) genes whose spatio-temporal transcription dynamics can be clearly differentiated throughout the 80 minute time course. *JAK2* shows a leading edge in the PolII cascade that reaches the 3′ end by 80 minutes time point, whereas *COBLL1* shows a similar lagging edge.

To identify genes that respond rapidly to estradiol stimulus via direct binding *in cis* of liganded 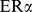 to estrogen response elements (EREs), we identified genes having 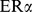 binding in their promoter region using ChIP-seq at 5 minutes after stimulus. Out of 17,218 genes in the annotated genome, we identified 5,553 genes (32%) that had at least one 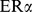 binding peak (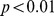, FDR corrected) within a 20 kb window of their annotated TSS. From the set of 699 estradiol induced genes, as defined by SPINLONG, 280 (40%) were associated with ER binding peaks within a 20 kb window from TSS (Supporting Information). The number of genes (280) with at least one 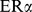 binding peak within 20 kb is significantly higher for the set of 777 genes identified by SPINLONG as compared to all annotated human genes (5,553 genes, 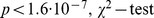).

### Survival associations of 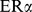 responder genes

Adjuvant endocrine therapy with selective anti-estrogens or with aromatase inhibitors is used to treat breast cancer patients with 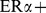 tumors [14]. Not all ER+ patients respond to these regimes and currently biomarkers able to predict the outcome of adjuvant therapy do not exist. We hypothesized that a fraction of genes driving resistance to 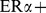 therapy may belong to early responsive genes to estradiol. Thus, we evaluated the survival association of the 777 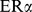 responsive genes identified by SPINLONG in The Cancer Genome Atlas (TCGA) breast cancer cohort [9]. The patients in the survival analysis were selected so that they corresponded to the characteristics of the MCF-7 cell line as follows. First, as menopause is a key clinical parameter in deciding therapeutic regimen [15], we selected patients who were either annotated as post-menopausal in TCGA or were older than 55 at the diagnosis and thus likely post-menopausal. Second, the ER pathway can be activated by HER2 in the absence of estrogen and HER2+/ER+ tumors are reported to be resistant to endocrine therapy [8]. Accordingly, we selected patients with known HER2- status. Using these criteria our survival analysis consisted of 150 ER+/HER2- primary breast cancer samples from post-menopausal patients.

**Figure 4:**
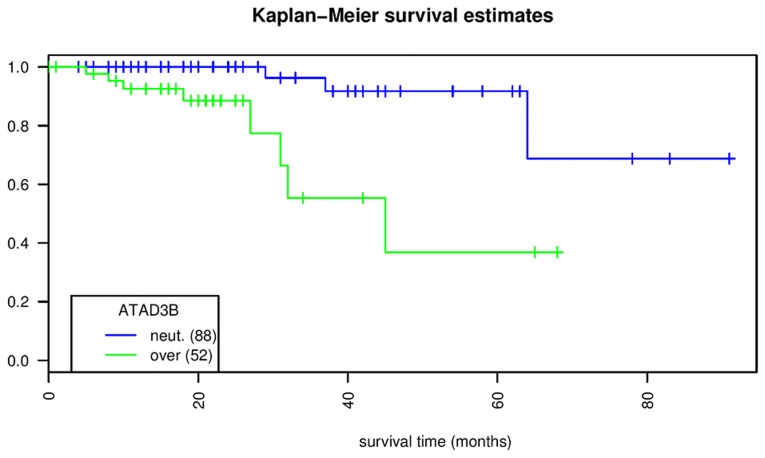
Kaplan-Meier survival plot comparing TCGA patients with over-expression (52 patients) or neutral expression (88 patients) of *ATAD3B*. Vertical ticks represent censoring events. The X and Y axes represent follow-up time in months and the percentage of survival, respectively. The associated log-rank p-value is 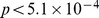.

Kaplan-Meier analysis with log-rank test resulted in 19 genes that have a survival effect with a nominal 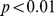 out of 777 early 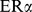 responsive genes. The genes are shown in Table S1 and all Kaplan-Meier curves in Figures S1, S2, S3, S4, S5, S6, S7, S8, S9, S10, S11, S12, S13, S14, S15, S16, S17, S18, S19. The gene ATPase family, AAA domain containing 3B (*ATAD3B*), which is a c-MYC and myogenin target gene and is expressed in highly proliferative tissues [16], had the lowest survival p-value (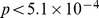). The Kaplan-Meier curve for *ATAD3B* is illustrated in Figure 4. Five years after diagnosis, less than 40% of the patients with over-expression of *ATAD3B* are alive, whereas 90% of patients without *ATAD3B* over-expression are alive at this point. At the mRNA level as measured by RNA-seq, *ATAD3B* is expressed in all time points and overexpressed in time points 160–1280 minutes compared to the baseline (data not shown).

In order to find out whether the survival association of *ATAD3B* expression is correlated with disease progression, we performed a Cox survival analysis for *ATAD3B* with tumor stage as a control variable. This analysis indicates that *ATAD3B* has predictive power independent from tumor stage (Cox 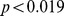).

### Estimating the propagation speed of PolII in E2 responder genes

The PolII transcription process consists of initiation, elongation and termination phases, with elongation occurring at 0.5–4 kb/min [4], [17]. There is evidence, however, that under optimal conditions, PolII speed can exceed 50 kb/min [18]. Through the time-series measurement of PolII progression in conjunction with the SPINLONG analysis, it is possible to estimate the speed of PolII on 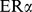 responsive genes. The PolII cascade forms a leading (induced genes) or lagging (repressed genes) edge, whose position is obtained from SPINLONG segment lengths. Comparing the locations of these edges at each time point gives lower bound estimates for propagation speed, *i.e.*, a large difference in edge locations in neighboring time points indicates high speed.

We observed that the transcription rate of PolII correlates strongly with gene length (Figure S20), with long genes transcribed at higher rates. The majority of estimates were between 1 and 10 kb/min, with some estimates exceeding 10 kb/min. High speed estimates were primarily obtained for induced genes. Early responding genes showed a rapid propagation of transcription across the gene body, suggesting rapid PolII elongation probably due to an open chromatin configuration in these genes [19]. While rapid propagation of PolII initiated transcription is in line with recent reports [18], the presence of multiple PolII cascades hinders estimation of PolII synthesis and elongation speed directly from sequencing data. Thus, the speeds reported here should be interpreted as the speed of information propagation.

Interestingly, we noted that genes with an 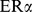 site within 20 kb of the TSS (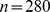) had a median transcription rate of 1,983 bp/min, which is significantly higher than the rate of 1,597 bp/min for genes (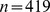) without an 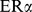 site (permutation test, 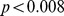). This effect is not explained by differing gene lengths in the two gene sets, as the gene lengths are comparable (permutation test, 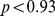).

## Discussion

We have developed a novel data-driven computational approach to facilitate the analysis of cell processes that are characterized by spatio-temporal signals. The large degree of freedom in defining patterns allows SPINLONG to scale to a variety of experimental designs where the user is able to formulate patterns to be identified within the data. Although the need to define the patterns *a priori* may place some conceptual load on the user, this approach offers a methodology to fuse hypothesis- and data-driven experimental designs. Furthermore, the segmentation-based paradigm allows clear visualization of the identified patterns and input data, which facilitates interpretation of results.

Methodologically, SPINLONG is a machine learning algorithm that can be used for classification, such as to determine whether a gene is induced, repressed or neither, but also for “sequence labeling” tasks, such as dividing a gene into flanking regions of PolII activity and inactivity. In genomic data analysis, SPINLONG shares some similarities with ChIP-seq peak detection algorithms (*e.g.*, [20]–[22]), as both aim to define spatial patterns in quantified DNA data. The unique feature of SPINLONG is that it permits more complex patterns to be discovered than peaks in single samples, including temporal patterns in multi-marker time-series experiments. This flexibility is achieved through the use of simulated annealing [23], which allows for complex objective functions, as well as linear algebra constraints for expressing relationships between samples and spatial segments of target regions.

When we applied SPINLONG to a time-series data from MCF-7 breast cancer cell line after 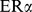 stimulus, we identified 777 early responsive genes. This set included several established 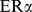 target genes, such as *XBP1*, *GREB1* and *CCND1*. Recently, Hah and colleagues used GRO-seq in MCF-7 cells and listed 3,098 estradiol response protein-coding transcripts [24]. For SPINLONG identified induced genes, 127 out of 699 (18%) genes were also induced in the GRO-seq dataset (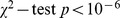 assuming a total of 10,000 profiled genes), whereas for repressed genes 31 out of 78 (40%) were found in both analyses (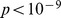). The main reason for the relatively low number of overlapping genes is that SPINLONG used more stringent criteria, which require that the spatio-temporal profiles of a gene across all time points are consistent. Additionally, there are differences in sequencing depths: Hah *et al.* used 6–10 million mapped reads per sample whereas we used approximately 100 million. The consequence of varying sequencing depths is illustrated in Figures S21 and S22 for the genes *XBP1* and *CCND1*, which are classical 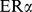 responsive genes and belong to the top five in the SPINLONG results. *CCND1* belongs to the list of the 3,098 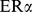 responsive genes in [24] and it has relatively good coverage. However, *XBP1* is not detected as 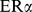 responsive genes in [24] and this is clearly due to insufficient sequencing depth. In the SPINLONG analysis, *XBP1* is the strongest candidate for an induced gene with a score of 1.00, and its expression has been reported to be 11-fold induced in MCF-7 cells after estradiol stimulus [25].

In addition to recent PolII profiling-based efforts, ER responsive genes have been investigated using microarrays, RNA sequencing and low-throughput methods. These resources include ERGDB [26], ERTargetDB [27] and Cicatiello *et al.* [28]. The numbers of ER responsive genes in these studies were 1,208 (ERGDB), 1,570 (ERTargetDB) and 1,150 (Cicatiello *et al.*). We compared these three and SPINLONG identified ER responsive gene sets using a set overlap analysis (Figure S23). The overlap between any pairs of the four ER responsive gene sets was small and there were only 13 genes that were common to all four sets. One reason for small concordance is experimental factors. For example, the PolII-based SPINLONG analysis captures early response genes, whereas mRNA profiling at 24 hours includes secondary responses and misses transient early responses.

Our results show that only 40% of 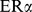 early responsive genes have 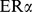 binding within 20 kb from TSS at 5 min after estradiol stimulus. This result is not unexpected as it is known that liganded 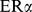 can alter gene expression by directly binding to estrogen response elements (genomic activation), or by non-genomic activation via the MAPK cascade or mitochondria [29]. To further characterize the biological processes for 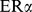 responsive genes with and without 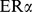 binding within 20 kb, we conducted Gene Ontology (GO) enrichment analysis. The GO enrichment analysis (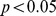, FDR-corrected) for induced genes having an 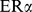 binding site produced only four enriched GO terms (Table S2). These terms are unspecific and their significance in 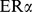 mediated responses is elusive. In contrast, genes without 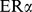 binding produced 71 enriched GO terms (Table S3), including *translational initiation* and *G1/S transition of mitotic cell cycle*, which are biological processes known to be regulated by 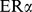 activation.

Kaplan-Meier analysis with 150 primary breast cancer tumors from TCGA resulted in 19 early 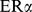 responsive genes that have survival association (Table S1). *ATAD3B* had the strongest statistically significant survival association. Its over-expression has been associated with chemoresistance in several cancers [16]. Our results indicate, for the first time, that over-expression of *ATAD3B* is also associated with significantly lower survival of post-menopausal breast cancer patients with ER+/HER2− tumors. Interestingly, there are no 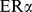 binding sites within 20 kb of the TSS for *ATAD3B*. This suggests that *ATAD3B* is activated through an 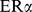 mediated non-genomic MAPK pathway regulated transcription factors, such as myogenin and c-Myc. To verify the survival association of *ATAD3B*, we conducted a Kaplan-Meier analysis in two other breast cancer cohorts, Miller *et al.* [30] and Pawitan *et al.* [31], having 130 and 159 primary breast cancer tumor samples with expression measurement for *ATAD3B*, respectively. The results were consistent with the TCGA analysis, with higher *ATAD3B* expression leading to poor survival (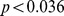 for Miller *et al.* and 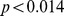 for Pawitan *et al.*; Figures S24, S25).

The significance of 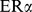 binding in breast cancer patient clinical outcome has been recently reported [3], whereas the role of the non-genomic pathway has gained less attention. Our results indicate that 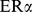 responsive genes with direct 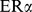 binding have different function and median transcription speed than the 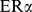 responsive genes without 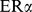 binding. The GO analysis suggests that the non-genomic 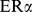 activation is crucial in exerting the transcriptional responses due to estradiol stimulus though we cannot exclude the possibility of direct distal 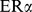 regulation. Furthermore, our results point to the direction that the non-genomic pathway, via *ATAD3B*, may play a role in resistance to anti-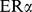 therapeutics in breast cancer. Accordingly, inhibition of the MAPK pathway leading to inactivation of myogenin or Myc could lead to down-regulation of *ATAD3B* and thus provide a putative therapeutic target for post-menopausal ER+/HER2− breast cancer patients. Even though more work is needed to firmly establish the role of *ATAD3B* in this subtype of breast cancer, our results demonstrate that SPINLONG is capable of producing experimentally testable hypotheses from large-scale deep sequencing data.

SPINLONG can be customized according to research hypotheses, as demonstrated here for the propagation of PolII on a gene. This, together with a computationally fast implementation that allowed to process 1.2 billion data points in 8 hours, makes SPINLONG a strong method to analyze multi-level deep sequencing data. In addition to ChIP-seq data, SPINLONG can be applied to data produced by other deep sequencing technologies, such as GRO-seq data, provided that the deep sequencing depth is sufficiently deep (greater than 100 million reads per sample) in order to identify patterns reliably. In summary, SPINLONG is a widely applicable novel computational method that integrates multiple levels of deep sequence data to produce experimentally testable hypotheses.

## Materials and Methods

### SPINLONG

SPINLONG is implemented as a freely available command line tool as well as a component to the Anduril workflow framework [32] with a comprehensive user guide at http://csbi.ltdk.helsinki.fi/spinlong.

#### Data import and preprocessing

In the sequencing data import step, the genome is divided into fixed length bins of size 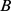 nucleotides and short read counts in each bin are computed for each sample. Default 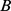 is 300 nucleotides; a relatively large bin size is used to reduce spatial noise. Each sample is normalized for differences in sequencing depth by making the sum of bin counts constant across the genome. Optionally, a control sample is used to control for local variation in ChIP-seq by subtracting control bin counts from each non-control sample bin count.

After data import, we focus on a pre-defined genomic region 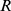, which is a chromosomal interval overlapping with a number of bins. To reduce spatial variation in bins, a median filter with a window of nine bins is applied within 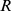. To reduce noise caused by low signal, background level of the region 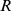 is estimated as 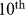 percentile in the bin count distribution within 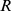 and this level is subtracted from all bins in the region. Negative counts are clamped to zero. As a result of preprocessing, we obtain an array of normalized bin counts for each sample.

#### Pattern matching

The core of the SPINLONG approach is pattern matching by score optimization. A pattern encodes a hypothesis, such as “transcription of a gene is initiated close to its starting site after stimulus and transcription progresses from 5′ to 3′ direction.” A score indicates how well the bin counts of the current region match the pattern. The user can define the expected spatial arrangements of bin counts in the samples, a scoring scheme and optional constrains for optimization.

Pattern matching is done in the context of a genomic region and the pattern is evaluated against all defined regions, such as all genes in a genome. A pattern divides the bins of each sample into *segments*, which are non-overlapping sub-intervals of 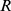. Each segment is annotated with a label that describes the expected bin count class of the segment. The classes 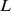 and 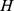 denote “low” and “high” bin counts, respectively, and 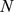 (ignored) denotes that the segment does not participate in scoring. Together, segments and their classes describe the expected spatial distribution of bin counts within samples. The user can define the number and classes of segments as well as their constraints, while their lengths are assigned in the optimization step.

Formally, the segments of a pattern are denoted as 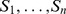 and their assigned lengths (in nucleotides) as 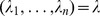, where 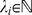. The lengths 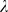 are assigned by a score-maximizing optimization process. Segment lengths are constrained by structural and user-defined constraints. An example of a structural constraint is the requirement that the segments in a sample must cover all the bins of the sample: 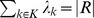, where 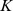 denotes segments belonging to the specific sample and 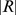 denotes the length of the current region. Optional user-defined constraints are used to fine tune and guide the optimization process. These constraints include segment length boundaries 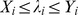 and global constraints 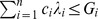 (where 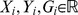). Global constraints allow defining relationships within and between samples. These allow, for instance, expressing constraints between time points and distinct ChIP targets.

#### Pattern scoring

The pattern scoring step evaluates each genomic region against the hypothesis and guides the optimization step. The result of the pattern scoring is the primary outcome of SPINLONG.

Pattern scoring uses assigned lengths (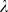) together with bin counts and segment classes to obtain a composite score. Patterns are scored in a two-step fashion, where individual segments are scored first and then combined into a composite score. A general scheme for segment scoring is to assign a score for each segment 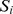 using the current length 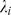 and state information (denoted 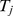) maintained by the optimizer. We denote by *segment scorer* a data structure that is responsible for assigning scores to specific segments and maintains its dynamic state information 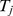.

All segments belonging to the same sample are scored with the same scorer, but there may be several independent scorers for independent sets of samples. Samples for distinct markers, such as PolII and H3K4me3, use separate scorers because their data ranges are different and segment scorers assume a common range for all associated samples. Segment score for a segment 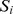 and scorer index 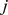 is denoted 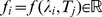.

#### Segment scoring using HMM

Segment scoring is done using 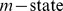 Hidden Markov Models (HMM) [33]. Bin counts are considered as Gaussian observations generated by 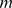 states of the model. The default 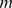 is two. The model is trained from all bins assigned to the scorer using the Baum-Welch method. HMM states are ordered based on the expected values of the Gaussian distributions in ascending order. HMM states of each bin are obtained using the Viterbi algorithm. HMM processing is done using JaHMM (http://code.google.com/p/jahmm/). Scorer state 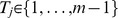 is the number of HMM states which are considered as low (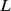) class: this allows to use 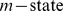 HMMs in the context of binary segment classes. The segment score is the proportion of bins whose HMM state matches the segment class: 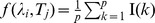, where
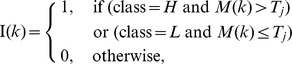


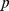
 is the number of bins in the segment and 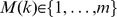 is the HMM state of bin 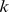.

#### Composite scores

Composite pattern scores 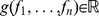 are obtained from segment scores by interpreting them as fuzzy truth values [34]. We derived four basic composite scoring methods as follows. Composite scores 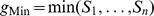 and 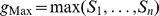 correspond to logical conjunction and disjunction, respectively. Of these, 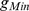 is generally more relevant for pattern matching: it means that “all segments match their respective class labels”. Between these extremes, 
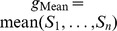
 includes information from all segment scores into the composite value, which is a desirable feature for guiding the optimization process. Combining 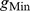 and 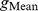, we obtain 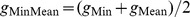 that includes a conjunctive component while also taking into account all score values. In addition to these four methods, users can define custom composite score functions using nested *min*, *max* and weighted *mean* functions.

#### Nonlinear optimization

Simulated annealing (SA) [23] is used to maximize composite score 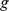. Valid solution vectors for SA are 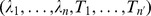 that satisfy linear constraints for segment lengths (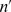 is the number of scorers). In the SA step, either the vector 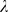 or one of 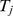 is modified. Segment lengths are modified using elementary operation vectors 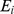 so that 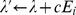, where 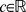. Elementary operations are obtained from basis vectors of the linear space corresponding to valid vectors 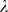: if 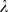 satisfies constraints, 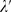 also does.

### Experiments with MCF-7 cell line

The deep sequencing data are available at http://csblsynergy.fimm.fi/. In addition to the data used in this work, the repository also contains MBD (methyl binding domain) ChIP-seq data. The database includes the protocol description for each of the experiments, the short reads and the aligned data files for each of the time points assayed.

#### Preparation of MCF-7 cells

The MCF-7 human breast cancer cell line originates from a 69-year old Caucasian woman and is estrogen receptor (ER) positive, progesterone positive (PR) and HER2 negative. Here MCF-7 cells (a clonal isolate kindly provided by Prof. Edison Liu, Jackson Laboratories, Maine, USA) were grown in 15 cm plates to 80% confluency. Plates were then washed 2 times with PBS and overlaid with 20 ml of phenol-red free high glucose DMEM (Gibco) containing 2% charcoal stripped FCS (Sigma). After 24 hours of incubation, the cells were again washed with PBS and fresh media containing 2% charcoal stripped FCS was added. This process was repeated over a three day period to generate cells devoid of estradiol signaling. The time course (10, 20, 40, 80, 160, 320, 640 and 1280 min) was initiated by replacing media with prewarmed media containing 10 nM E2. In addition, an untreated sample was included in the experiment as a zero time point.

#### ChIP-seq protocols and methods

For chromatin immunoprecipitation, cells were fixed for 10 minutes at room temperature by the addition of formaldehyde to a final concentration of 1%, after which glycine was added to a concentration of 100 mM. Cells were then washed twice with PBS and collected into 2 ml of lysis buffer (150 mM NaCl, 20 mM Tris pH 8.0, 2 mM EDTA, 1% triton X-100, protease inhibitor [complete EDTA free, Roche, 04 693 132 001], 100 mM PMSF). The lysate was sonicated for 3×30 seconds using a Branson ultrasonicator equipped with a microtip on a power setting of 3 and a duty cycle of 90%. Samples were cooled on ice between rounds of sonication. Alternatively, a Bioruptor sonicator was used (power high, 15 mins total, 30 s on 30 s off; total volume of sample – 1 ml) to fragment chromatin. In either case, the resulting sonicate was centrifuged at 4000×g for 5 minutes, an aliquot of 10% retained for input and the remaining material transferred to a fresh tube.

Four 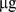 of anti-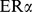 antibody (HC-20, rabbit polyclonal, Santa Cruz, sc-543), 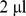 of anti-RNA Polymerase II antibody (AC-055-100, monoclonal, Diagenode, 001), 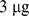 of anti-H3K4me3 antibody (pAb-MEHAHS-024, rabbit polyclonal, Diagenode, HC-0010) and 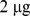 anti-Histone H2A.Z (acetyl K4+K7+K11) antibody (ab18262, sheep polyclonal, Abcam, 659355) were added to the samples, which were then incubated overnight at 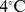 with rotation. Chromatin antibody complexes were isolated, either by addition of 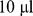 of protein G labeled magnetic beads (Millipore Pureproteome protein G magnetic beads, LSKMAGG10) prewashed in lysis buffer or with 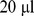 protein A/G beads (Santa Cruz). Afterwards, the complexes obtained with protein G magnetic beads were washed three times with lysis buffer, then reverse crosslinked in 0.5 ml 5 M guanidine hydrochloride, 20 mM Hepes, 30% isopropanol, 10 mM EDTA for a minimum of 4 hours at 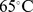. Recovered DNA was then purified using a Qiaquick spin column and eluted in 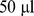 of 10 mM Tris pH 8.0. Where protein A/G beads were used, the complexes were washed sequentially with three different buffers at 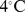: two times with solution of composition 0.1% SDS, 0.1% DOC, 1% Triton, 150 mM NaCl, 1 mM EDTA, 0.5 mM EGTA, 20 mM HEPES pH 7.6, once with the solution as before but with 500 mM NaCl, once with solution of composition 0.25 M LiCl, 0.5% DOC, 0.5% NP-40, 1 mM EDTA, 0.5 mM EGTA, 20 mM HEPES pH 7.6 and two times with 1 mM EDTA, 0.5 mM EGTA, 20 mM HEPES pH 7.6. A control library was generated by sequencing input DNA (non-ChIP genomic DNA). Immunopurified chromatin was eluted with 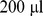 of elution buffer (1% SDS, 0.1 M NaHCO3), incubated at 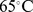 for 4 h in the presence of 200 mM NaCl, isolated using a Qiaquick spin column and eluted in 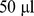 of 10 mM Tris pH 8.0.

To compute the associations between 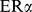 binding peaks and genes, we used an exponential distance model with a score threshold of one and unit intensity for all peaks [35]. Peak detection was done using MACS [20].

#### RNA-seq protocols

Total RNA was isolated using Trizol (Invitrogen) according to the manufacturer's recommendations, followed by DNazol treatment (QIAGEN). 100–250 ng total RNA was subjected rRNA depletion with the Ribo-Zero rRNA Removal Kit (Human/Mouse/Rat; cat. no. RZH110424). The rRNA depleted sample was purified by ethanol precipitation. mRNA was fragmented by hydrolysis (5× fragmentation buffer: 200 mM Tris acetate, pH 8.2, 500 mM potassium acetate, and 150 mM magnesium acetate) at 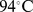 for 90 sec and then purified (RNeasy MinElute Cleanup Kit, QIAGEN). cDNA was synthesized using 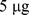 random hexamers using Superscript III Reverse Transcriptase (Invitrogen). ds-cDNA synthesis was performed in second strand buffer (Invitrogen) according to the manufacturer's recommendations and then purified (MinElute Reaction Cleanup Kit, QIAGEN). ds-cDNA was prepared for Illumina sequencing according to the manufacturer's protocols (Illumina). Briefly, ds-cDNA fragments were subject to sequential end repair and adaptor ligation. ds-cDNA fragments were subsequently size selected (approx. 300 base pair [bp]). The adaptor-modified DNA fragments were amplified by limited PCR (14 cycles). Quality control and concentration measurements were made by analysis of the PCR products by electrophoresis (Experion, BioRad) and by fluorometric dye binding using a Qubit fluorometer with the Quant-iT dsDNA HS Assay Kit (Invitrogen, Q32851) respectively. Cluster generation and sequencing-by-synthesis (36 bp) was performed using the Illumina Genome Analyzer IIx (GAIIx) according to standard protocols of the manufacturer (Illumina).

#### Alignment and preprocessing of sequencing data

To align all reads, we used the GMS_map software (version 3.2.1) on a Genomatix Mining Station. The reference genome used was human hg19 (NCBI build 37). The software uses a seed-based approach to align reads. Mapping a read to a reference sequence involves two steps. In the first step, seeds for potential mapping positions in the target sequence are identified via a mapping library built of short unique subwords from the reference sequence. In the second step, alignments of the complete sequence read to the previously identified positions in the reference sequence are calculated. Results are ranked by their alignment score. We used the ‘deep’ seed search option allowing for point mutations during seed search. The overall alignment quality threshold was set to 92%, allowing for at most two point mutations. Uniquely and multiply matching reads meeting these thresholds were provided in BED and indexed BAM format.

#### SPINLONG patterns for identifying PolII activity regions

To estimate PolII activity start and end sites, we combined the time points 0, 10, 20, 40 and 80 min together into a “pseudo-sample” that contains the maximum of bin counts in these time points. This was done individually for each marker (PolII, H3K4me3 and H2A.Z). These pseudo-samples contain information from all time points but also simplify pattern formulation by including only one (pseudo-) sample for each marker, instead of five samples for individual time points.

All pseudo-samples were divided into three segments with classes 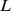 (low), 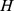 (high) and 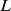, which correspond to the gene upstream, body and downstream, respectively. We thus obtained nine segments, denoted 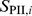 (PolII), 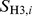 (H3K4me3) and 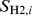 (H2A.Z), where 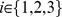. Based on the assumption that histone markers colocalize with PolII in promoters, histone upstream segments were synchronized with PolII using global constraints 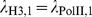 and 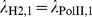: that is, the leftmost segments in each sample must be identical in length for all valid solutions 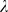.

Composite scoring was based on 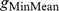, but takes into account genes for which one or both histone markers are weakly present. The score function is 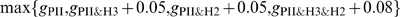, where 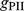 is 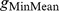 taking only PolII into account, 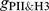 is 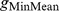 taking PolII and H3K4me3 into account, and similarly for other components. Offsets of 0.05 and 0.08 were chosen to bias the scorer to use all markers when possible.

#### SPINLONG patterns for identifying 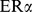 responder genes

The assumption behind identifying genes whose activity levels are increased after a stimulus is that initially and without stimulation such a gene is transcribed with a low rate and thus there is little PolII activity across the gene. Similarly, we assume that genes whose activity is repressed after a stimulus initially show high PolII activity, which is decreased after a stimulus. For genes that are activated due to a stimulus, the PolII complex binds to the genomic regions at the transcription start site (TSS) of a responder gene. This is indicated by an increased number of reads in the 5′ end of the gene. These assumptions are used to form hypotheses used in SPINLONG.

Identification of estrogen responder genes is divided into identifying induced and repressed genes. For this analysis, PolII data were used at time points 0, 10, 20, 40 and 80 min; in addition, time points 160 and 640 min were used to compute a pseudo-sample (see below). The hypothesis for induced genes is that they show low signal (class 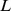) in 0 time point and a progressively elongating high signal (class 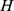) in later time points that represents a region of PolII binding. The 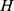 class segment at time point 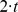 is expected to be approximately two times longer than at time point 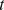, since time intervals increase geometrically and PolII elongation is assumed to be constant. This is encoded as a global linear constraint in the pattern, with the relaxation that length at 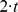 must be at least 1.5 longer than at 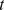.

Patterns for induction are named *inducedN*, where 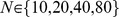 is the time point at which the gene is fully occupied with PolII, *i.e.*, the 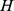 class segment covers the gene. The pattern *induced80* captures long genes that are not completely transcribed at last time point. Repressed genes show similar behavior but with classes 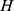 and 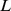 swapped; these patterns are named *repressedN*.

The segment configuration of each pattern is visible in the result visualizations (Figures 2 and 3 and result WWW site) and in SPINLONG pattern XML files (Supporting Information). Scoring is done using 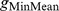. Genomic regions 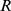 that are used as the search area are obtained from the predictions of PolII activity regions above, or from pre-defined Ensembl gene regions when the score in the activity prediction analysis is below 0.60. To allow the optimizer to further fine-tune the search area, these regions are extended by 25% in 5′ and 3′ directions and a pseudo-sample containing the maximum bin count of the time points 0, 10, 40, 160 and 640 min is included.

#### SPINLONG patterns for PolII propagation speed

Estimation of PolII propagation speed is done using the segment lengths obtained in the identification of estrogen responsive genes above. For the patterns *induced10* and *repressed10*, speed lower bound is estimated as 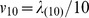 nt/min, where 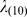 is the length of class 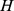 PolII segment at 10 minutes. For the patterns *induced20* and *repressed20*, two speed estimates were computed as 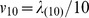 nt/min and 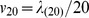 nt/min, where 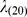 is the length of class 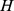 segment at 20 minutes. The minimum of these velocities gives the lower bound for the speed estimate. For 40 and 80 minute time points, lower bounds are computed in similar fashion using additionally 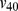 and 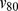.

#### Survival analysis

Survival analyses were conducted with the Anduril framework [32]. In all analyses, death from breast cancer was used as the event indicator. For the TCGA cohort [9], patients having ER+, HER2− and either post-menopausal status or age 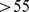 were selected. In addition, 65 healthy tissue samples were used as controls. For each gene, patients were divided into groups having low, high or stable expression. This was done by computing fold changes for each tumor and comparing them to a threshold based on the distribution of the fold changes. That is, let 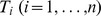 and 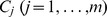 be 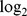 expression values for 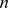 tumors and 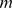 controls for a specific gene. Then, a median of control samples 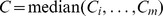 was computed. Sample-specific fold changes are 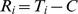 and their standard deviation is 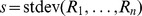. Now, a tumor was classified into the low (high) group for this gene if 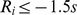 (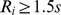) and into the stable group otherwise.

In the Cox regression model for *ATAD3B* we used TNM tumor stage as a control variable [36]. TNM tumor stage is determined by the size of the tumor (T), lymph node involvement (N) and whether the cancer has metastasized (M). There are five stage categories from which 0 represents non-invasive cancer and IV means an invasive cancer that has metastasized to distant organs. For instance, a stage I tumor is an invasive cancer where the tumor is small (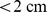) and which has not metastasized. Here we excluded stage 0 and combined subcategories for stage II (IIa and IIb) as well as for stage III (IIIa, IIIb and IIIc). In the Cox model, tumor stage is encoded as stage I = 1, II = 2, III = 3 and IV = 4.

The results published here are in part based upon data generated by The Cancer Genome Atlas pilot project established by the NCI and NHGRI. Information about TCGA and the investigators and institutions who constitute the TCGA research network can be found at http://cancergenome.nih.gov. The TSP study accession number in the database of Genotype and Phenotype (dbGaP) for the TCGA study used here is phs000569.v1.p7.

In the first *ATAD3B* validation cohort, Miller *et al.* [30], control samples and HER2 status were not available so patients having ER+ and age 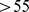 were selected. In the second validation cohort, Pawitan *et al.* [31], ER status or age were not reported so we selected all patients. Normalized microarray values were obtained using RMA [37]. In both cohorts, patients were grouped into “low” and “high” groups based on whether *ATAD3B* expression in the tumor sample was below or above expression median. A Kaplan-Meier analysis with the log-rank test was conducted based on these groupings for *ATAD3B*.

## Supporting Information

Figure S1 Kaplan-Meier survival plot comparing TCGA patients with overexpression (denoted 1), neutral expression (0) or underexpression (−1) of *GSTM4*. Expression groups with less than 20 patients are omitted. Vertical ticks represent censoring events. The X and Y axes represent follow-up time in months and the percentage of survival, respectively. The associated log-rank p-value is 2.304994e-03.
(PDF)

Figure S2 Kaplan-Meier survival plot comparing TCGA patients with overexpression (denoted 1), neutral expression (0) or underexpression (−1) of *GPR157*. Expression groups with less than 20 patients are omitted. Vertical ticks represent censoring events. The X and Y axes represent follow-up time in months and the percentage of survival, respectively. The associated log-rank p-value is 4.788661e-03.
(PDF)

Figure S3 Kaplan-Meier survival plot comparing TCGA patients with overexpression (denoted 1), neutral expression (0) or underexpression (−1) of *SLC37A4*. Expression groups with less than 20 patients are omitted. Vertical ticks represent censoring events. The X and Y axes represent follow-up time in months and the percentage of survival, respectively. The associated log-rank p-value is 3.828535e-03.
(PDF)

Figure S4 Kaplan-Meier survival plot comparing TCGA patients with overexpression (denoted 1), neutral expression (0) or underexpression (−1) of *EIF3B*. Expression groups with less than 20 patients are omitted. Vertical ticks represent censoring events. The X and Y axes represent follow-up time in months and the percentage of survival, respectively. The associated log-rank p-value is 7.649984e-03.
(PDF)

Figure S5 Kaplan-Meier survival plot comparing TCGA patients with overexpression (denoted 1), neutral expression (0) or underexpression (−1) of *ATAD3B*. Expression groups with less than 20 patients are omitted. Vertical ticks represent censoring events. The X and Y axes represent follow-up time in months and the percentage of survival, respectively. The associated log-rank p-value is 5.12124e-04.
(PDF)

Figure S6 Kaplan-Meier survival plot comparing TCGA patients with overexpression (denoted 1), neutral expression (0) or underexpression (−1) of *PPA2*. Expression groups with less than 20 patients are omitted. Vertical ticks represent censoring events. The X and Y axes represent follow-up time in months and the percentage of survival, respectively. The associated log-rank p-value is 1.315913e-03.
(PDF)

Figure S7 Kaplan-Meier survival plot comparing TCGA patients with overexpression (denoted 1), neutral expression (0) or underexpression (−1) of *ZNF275*. Expression groups with less than 20 patients are omitted. Vertical ticks represent censoring events. The X and Y axes represent follow-up time in months and the percentage of survival, respectively. The associated log-rank p-value is 8.437339e-03.
(PDF)

Figure S8 Kaplan-Meier survival plot comparing TCGA patients with overexpression (denoted 1), neutral expression (0) or underexpression (−1) of *VAPB*. Expression groups with less than 20 patients are omitted. Vertical ticks represent censoring events. The X and Y axes represent follow-up time in months and the percentage of survival, respectively. The associated log-rank p-value is 3.042901e-03.
(PDF)

Figure S9 Kaplan-Meier survival plot comparing TCGA patients with overexpression (denoted 1), neutral expression (0) or underexpression (−1) of *CBX8*. Expression groups with less than 20 patients are omitted. Vertical ticks represent censoring events. The X and Y axes represent follow-up time in months and the percentage of survival, respectively. The associated log-rank p-value is 4.160872e-03.
(PDF)

Figure S10 Kaplan-Meier survival plot comparing TCGA patients with overexpression (denoted 1), neutral expression (0) or underexpression (−1) of *BAG5*. Expression groups with less than 20 patients are omitted. Vertical ticks represent censoring events. The X and Y axes represent follow-up time in months and the percentage of survival, respectively. The associated log-rank p-value is 5.949707e-04.
(PDF)

Figure S11 Kaplan-Meier survival plot comparing TCGA patients with overexpression (denoted 1), neutral expression (0) or underexpression (−1) of *C6orf141*. Expression groups with less than 20 patients are omitted. Vertical ticks represent censoring events. The X and Y axes represent follow-up time in months and the percentage of survival, respectively. The associated log-rank p-value is 8.628732e-03.
(PDF)

Figure S12 Kaplan-Meier survival plot comparing TCGA patients with overexpression (denoted 1), neutral expression (0) or underexpression (−1) of *CTD-2526A2.1*. Expression groups with less than 20 patients are omitted. Vertical ticks represent censoring events. The X and Y axes represent follow-up time in months and the percentage of survival, respectively. The associated log-rank p-value is 3.254192e-03.
(PDF)

Figure S13 Kaplan-Meier survival plot comparing TCGA patients with overexpression (denoted 1), neutral expression (0) or underexpression (−1) of *PVR*. Expression groups with less than 20 patients are omitted. Vertical ticks represent censoring events. The X and Y axes represent follow-up time in months and the percentage of survival, respectively. The associated log-rank p-value is 5.386878e-03.
(PDF)

Figure S14 Kaplan-Meier survival plot comparing TCGA patients with overexpression (denoted 1), neutral expression (0) or underexpression (−1) of *ASPHD1*. Expression groups with less than 20 patients are omitted. Vertical ticks represent censoring events. The X and Y axes represent follow-up time in months and the percentage of survival, respectively. The associated log-rank p-value is 2.920611e-03.
(PDF)

Figure S15 Kaplan-Meier survival plot comparing TCGA patients with overexpression (denoted 1), neutral expression (0) or underexpression (−1) of *USP36*. Expression groups with less than 20 patients are omitted. Vertical ticks represent censoring events. The X and Y axes represent follow-up time in months and the percentage of survival, respectively. The associated log-rank p-value is 3.151684e-03.
(PDF)

Figure S16 Kaplan-Meier survival plot comparing TCGA patients with overexpression (denoted 1), neutral expression (0) or underexpression (−1) of *ADPRHL2*. Expression groups with less than 20 patients are omitted. Vertical ticks represent censoring events. The X and Y axes represent follow-up time in months and the percentage of survival, respectively. The associated log-rank p-value is 9.95715e-03.
(PDF)

Figure S17 Kaplan-Meier survival plot comparing TCGA patients with overexpression (denoted 1), neutral expression (0) or underexpression (−1) of *UBE2J2*. Expression groups with less than 20 patients are omitted. Vertical ticks represent censoring events. The X and Y axes represent follow-up time in months and the percentage of survival, respectively. The associated log-rank p-value is 1.76163e-03.
(PDF)

Figure S18 Kaplan-Meier survival plot comparing TCGA patients with overexpression (denoted 1), neutral expression (0) or underexpression (−1) of *ELF1*. Expression groups with less than 20 patients are omitted. Vertical ticks represent censoring events. The X and Y axes represent follow-up time in months and the percentage of survival, respectively. The associated log-rank p-value is 1.453872e-03.
(PDF)

Figure S19 Kaplan-Meier survival plot comparing TCGA patients with overexpression (denoted 1), neutral expression (0) or underexpression (−1) of *PMP22*. Expression groups with less than 20 patients are omitted. Vertical ticks represent censoring events. The X and Y axes represent follow-up time in months and the percentage of survival, respectively. The associated log-rank p-value is 6.4615e-03.
(PDF)

Figure S20 PolII elongation speed lower bound estimates compared to gene lengths. The gene set includes induced and repressed estradiol early response genes. Genes are colored accoring to the time point by which the leading or lagging end reaches the 3′ end; 80 minute time point includes genes for which the transition remains incomplete. For gene length, transcribed region lengths are used when this estimate is available; otherwise, lengths obtained from Ensembl are used.
(PDF)

Figure S21 Short read counts of *CCND1* in the GRO-seq experiments of [24], based on the Wiggle files in that publication. The screenshot is taken using IGV [38].
(PNG)

Figure S22 Short read counts of *XBP1* in the GRO-seq experiments of [24], based on the Wiggle files in that publication. The screenshot is taken using IGV [38].
(PNG)

Figure S23 Venn diagrams for ER responsive gene set intersections. Each number denotes the number of genes in the intersection. Data for the three external gene sets are from ERGDB [26], ERTargetDB [27] and Cicatiello *et al.* [28] (denoted Cic10; blue).
(PDF)

Figure S24 Kaplan-Meier survival plot comparing patients from [30] having above or below median expression of *ATAD3B*. Vertical ticks represent censoring events. Log-rank probability measure for the equality of these curves is 3.618236e-02.
(PDF)

Figure S25 Kaplan-Meier survival plot comparing patients from [31] having above or below median expression of *ATAD3B*. Vertical ticks represent censoring events. Log-rank probability measure for the equality of these curves is 1.418654e-02.
(PDF)

Table S1 Survival-associated genes in the TCGA cohort predicted to respond to estradiol stimulus by SPINLONG.
(PDF)

Table S2Gene Ontology enrichment for induced genes with an 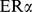 binding site.(PDF)

Table S3Gene Ontology enrichment for induced genes without an 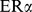 binding site.(PDF)
