# Supplementary figures and images for: Integrative Analysis of Deep Sequencing Data Identifies Estrogen Receptor Early Response Genes and Links ATAD3B to Poor Survival in Breast Cancer
Source: PLoS Comput Biol. 2013 Jun 20;9(6):e1003100. doi: 10.1371/journal.pcbi.1003100 (PMC3688481; doi:10.1371/journal.pcbi.1003100)

## Kaplan-Meier survival estimates

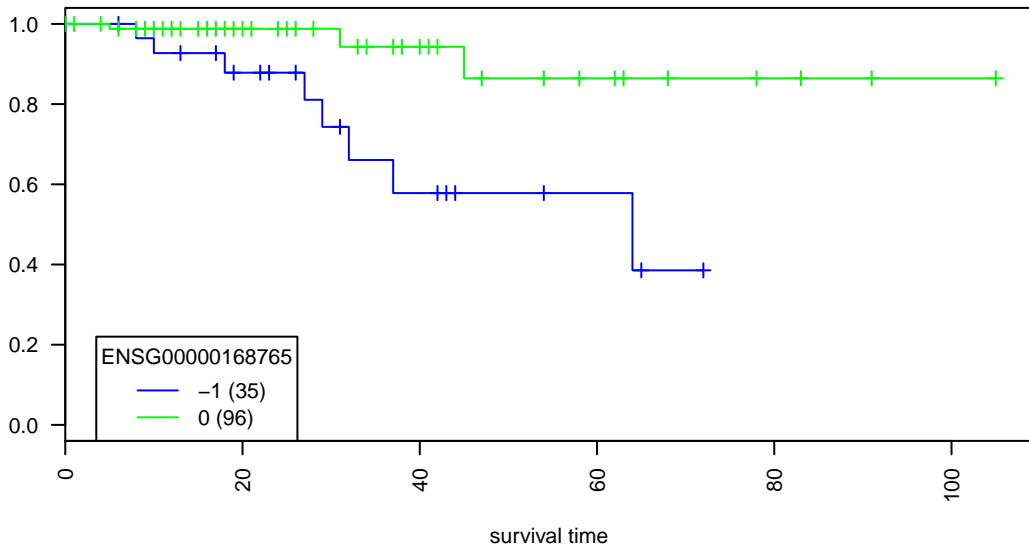

Supplement: Figure S1 — Kaplan-Meier survival plot comparing TCGA patients with overexpression (denoted 1), neutral expression (0) or underexpression (−1) of GSTM4. Expression groups with less than 20 patients are omitted. Vertical ticks represent censoring events. The X and Y axes represent follow-up time in months and the percentage of survival, respectively. The associated log-rank p-value is 2.304994e-03. (PDF) [file pcbi.1003100.s001.pdf]

## Kaplan-Meier survival estimates

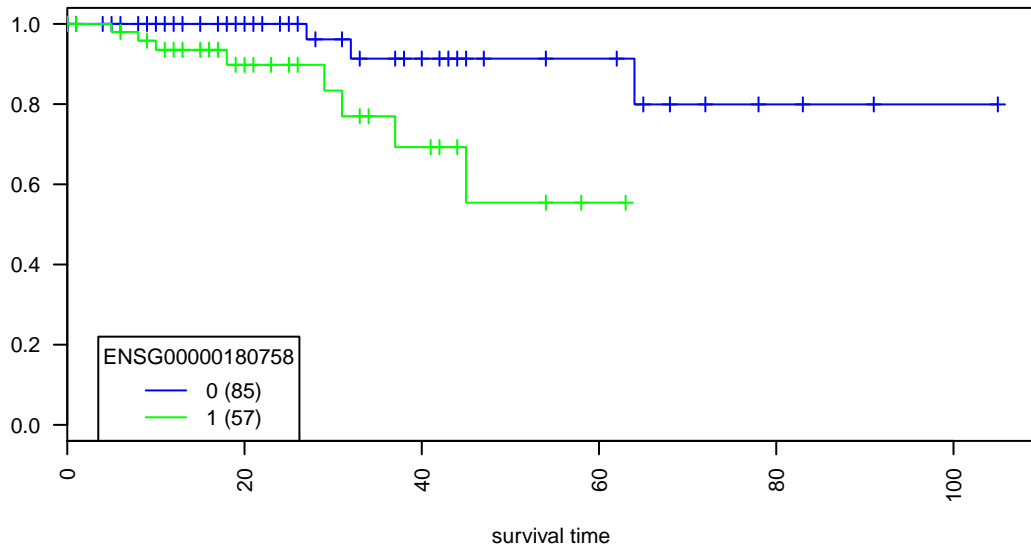

Supplement: Figure S2 — Kaplan-Meier survival plot comparing TCGA patients with overexpression (denoted 1), neutral expression (0) or underexpression (−1) of GPR157. Expression groups with less than 20 patients are omitted. Vertical ticks represent censoring events. The X and Y axes represent follow-up time in months and the percentage of survival, respectively. The associated log-rank p-value is 4.788661e-03. (PDF) [file pcbi.1003100.s002.pdf]

## Kaplan-Meier survival estimates

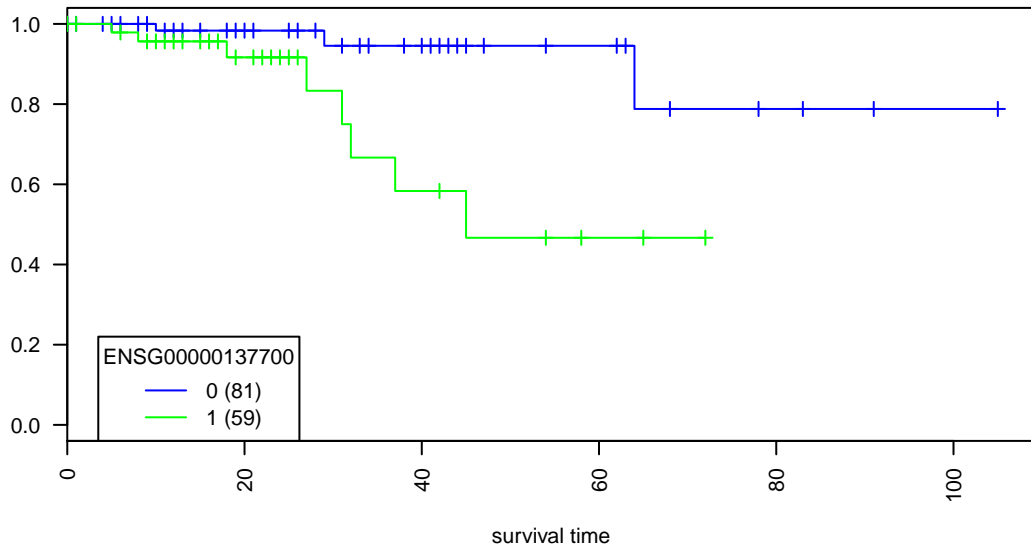

Supplement: Figure S3 — Kaplan-Meier survival plot comparing TCGA patients with overexpression (denoted 1), neutral expression (0) or underexpression (−1) of SLC37A4. Expression groups with less than 20 patients are omitted. Vertical ticks represent censoring events. The X and Y axes represent follow-up time in months and the percentage of survival, respectively. The associated log-rank p-value is 3.828535e-03. (PDF) [file pcbi.1003100.s003.pdf]

## Kaplan-Meier survival estimates

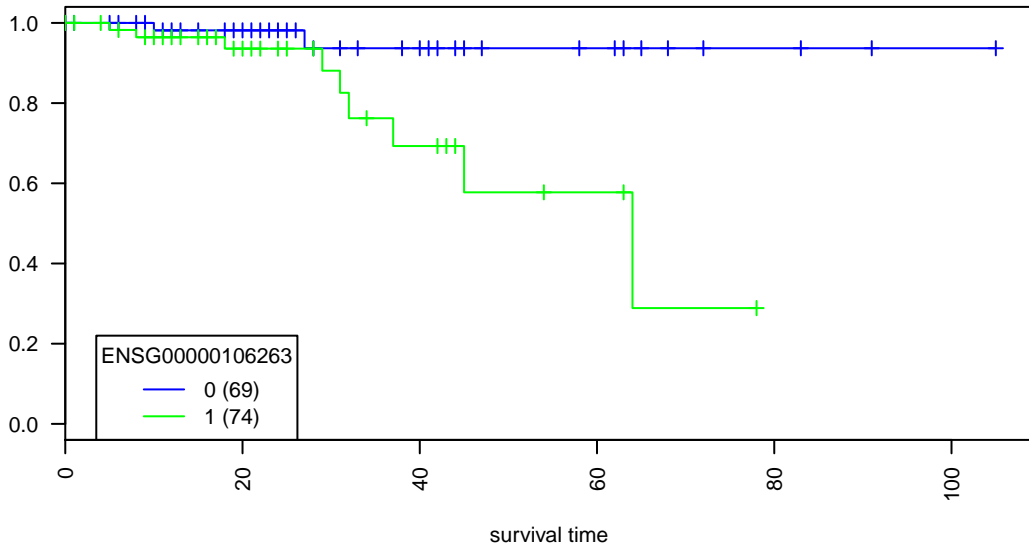

Supplement: Figure S4 — Kaplan-Meier survival plot comparing TCGA patients with overexpression (denoted 1), neutral expression (0) or underexpression (−1) of EIF3B. Expression groups with less than 20 patients are omitted. Vertical ticks represent censoring events. The X and Y axes represent follow-up time in months and the percentage of survival, respectively. The associated log-rank p-value is 7.649984e-03. (PDF) [file pcbi.1003100.s004.pdf]

## Kaplan-Meier survival estimates

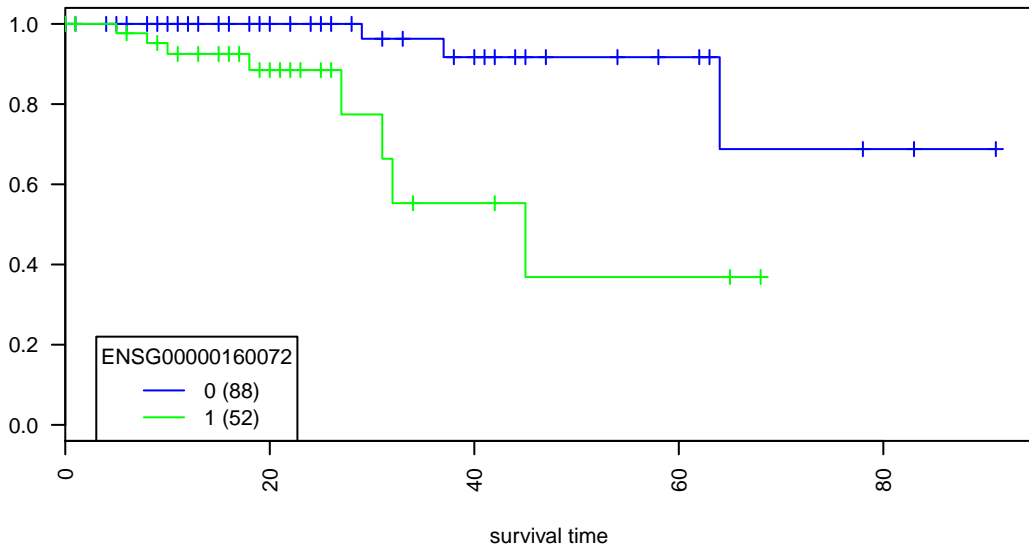

Supplement: Figure S5 — Kaplan-Meier survival plot comparing TCGA patients with overexpression (denoted 1), neutral expression (0) or underexpression (−1) of ATAD3B. Expression groups with less than 20 patients are omitted. Vertical ticks represent censoring events. The X and Y axes represent follow-up time in months and the percentage of survival, respectively. The associated log-rank p-value is 5.12124e-04. (PDF) [file pcbi.1003100.s005.pdf]

## Kaplan-Meier survival estimates

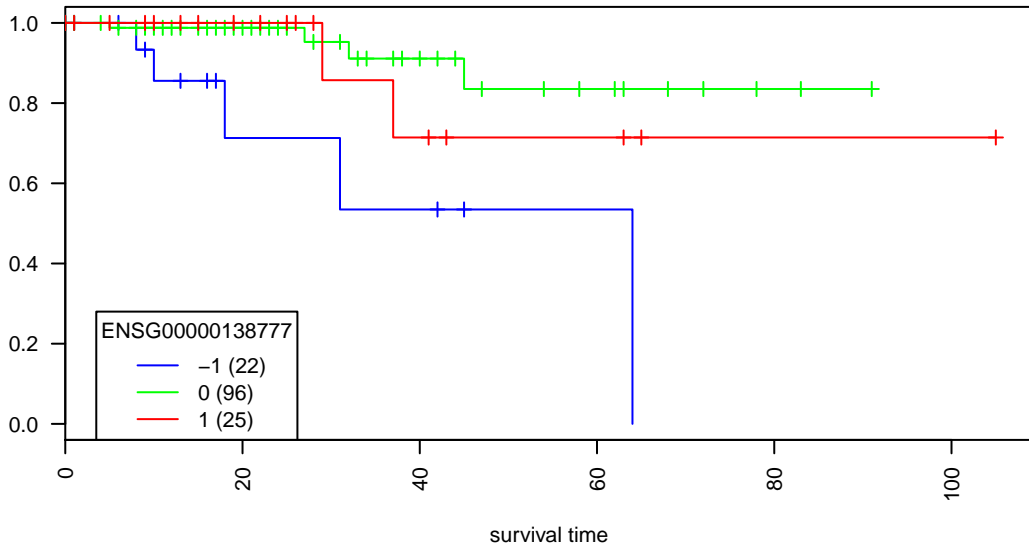

Supplement: Figure S6 — Kaplan-Meier survival plot comparing TCGA patients with overexpression (denoted 1), neutral expression (0) or underexpression (−1) of PPA2. Expression groups with less than 20 patients are omitted. Vertical ticks represent censoring events. The X and Y axes represent follow-up time in months and the percentage of survival, respectively. The associated log-rank p-value is 1.315913e-03. (PDF) [file pcbi.1003100.s006.pdf]

## Kaplan–Meier survival estimates

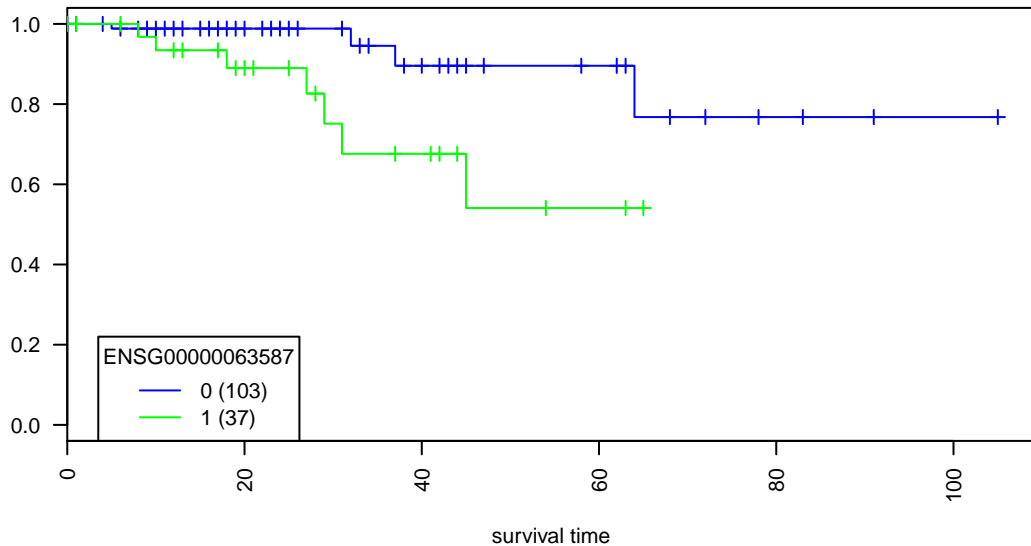

Supplement: Figure S7 — Kaplan-Meier survival plot comparing TCGA patients with overexpression (denoted 1), neutral expression (0) or underexpression (−1) of ZNF275. Expression groups with less than 20 patients are omitted. Vertical ticks represent censoring events. The X and Y axes represent follow-up time in months and the percentage of survival, respectively. The associated log-rank p-value is 8.437339e-03. (PDF) [file pcbi.1003100.s007.pdf]

## Kaplan–Meier survival estimates

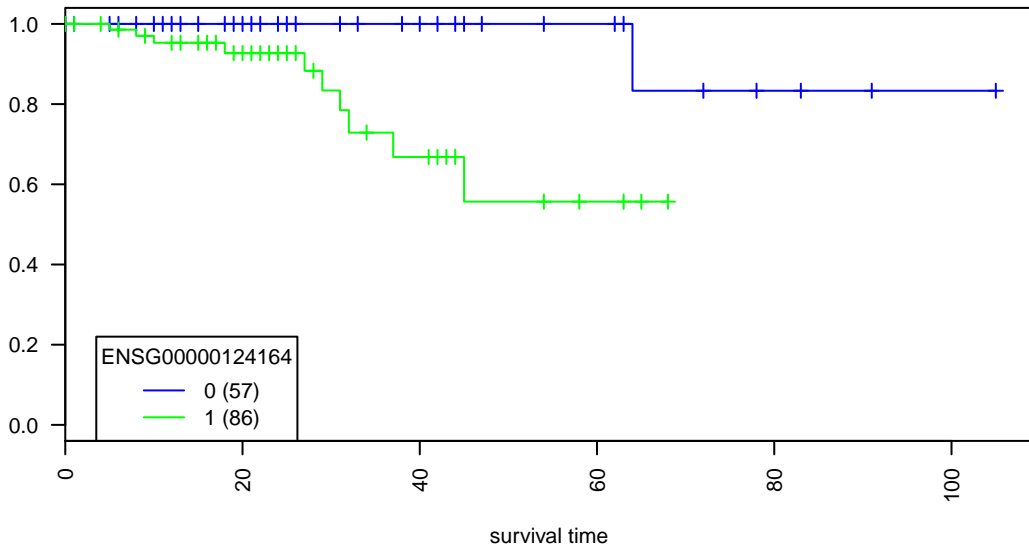

Supplement: Figure S8 — Kaplan-Meier survival plot comparing TCGA patients with overexpression (denoted 1), neutral expression (0) or underexpression (−1) of VAPB. Expression groups with less than 20 patients are omitted. Vertical ticks represent censoring events. The X and Y axes represent follow-up time in months and the percentage of survival, respectively. The associated log-rank p-value is 3.042901e-03. (PDF) [file pcbi.1003100.s008.pdf]

## Kaplan–Meier survival estimates

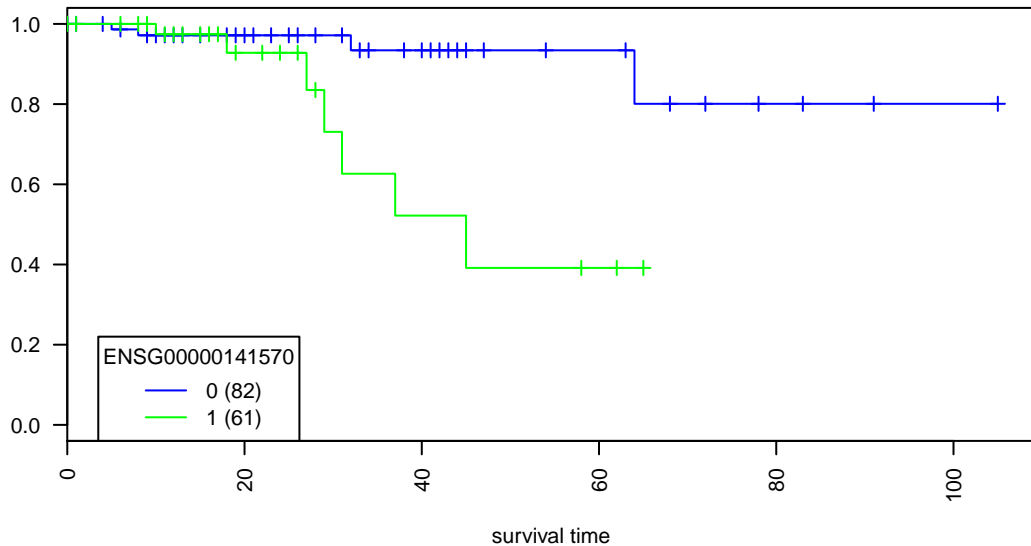

Supplement: Figure S9 — Kaplan-Meier survival plot comparing TCGA patients with overexpression (denoted 1), neutral expression (0) or underexpression (−1) of CBX8. Expression groups with less than 20 patients are omitted. Vertical ticks represent censoring events. The X and Y axes represent follow-up time in months and the percentage of survival, respectively. The associated log-rank p-value is 4.160872e-03. (PDF) [file pcbi.1003100.s009.pdf]

## Kaplan-Meier survival estimates

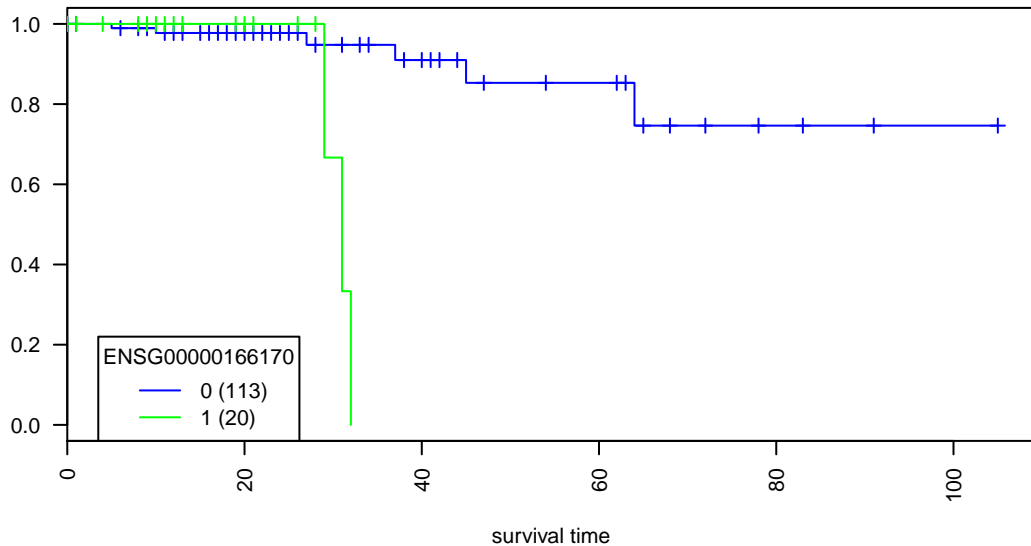

Supplement: Figure S10 — Kaplan-Meier survival plot comparing TCGA patients with overexpression (denoted 1), neutral expression (0) or underexpression (−1) of BAG5. Expression groups with less than 20 patients are omitted. Vertical ticks represent censoring events. The X and Y axes represent follow-up time in months and the percentage of survival, respectively. The associated log-rank p-value is 5.949707e-04. (PDF) [file pcbi.1003100.s010.pdf]

## Kaplan-Meier survival estimates

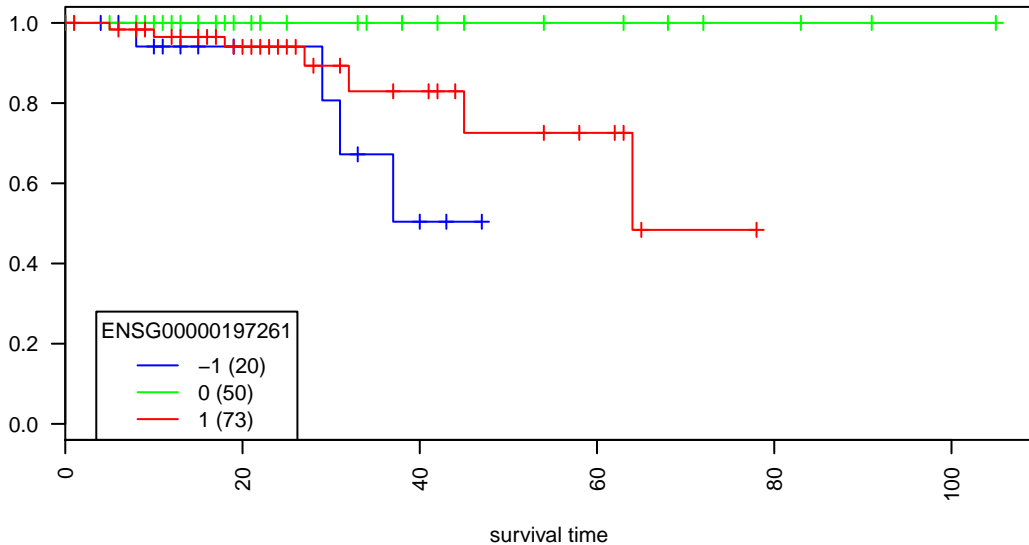

Supplement: Figure S11 — Kaplan-Meier survival plot comparing TCGA patients with overexpression (denoted 1), neutral expression (0) or underexpression (−1) of C6orf141. Expression groups with less than 20 patients are omitted. Vertical ticks represent censoring events. The X and Y axes represent follow-up time in months and the percentage of survival, respectively. The associated log-rank p-value is 8.628732e-03. (PDF) [file pcbi.1003100.s011.pdf]

## Kaplan-Meier survival estimates

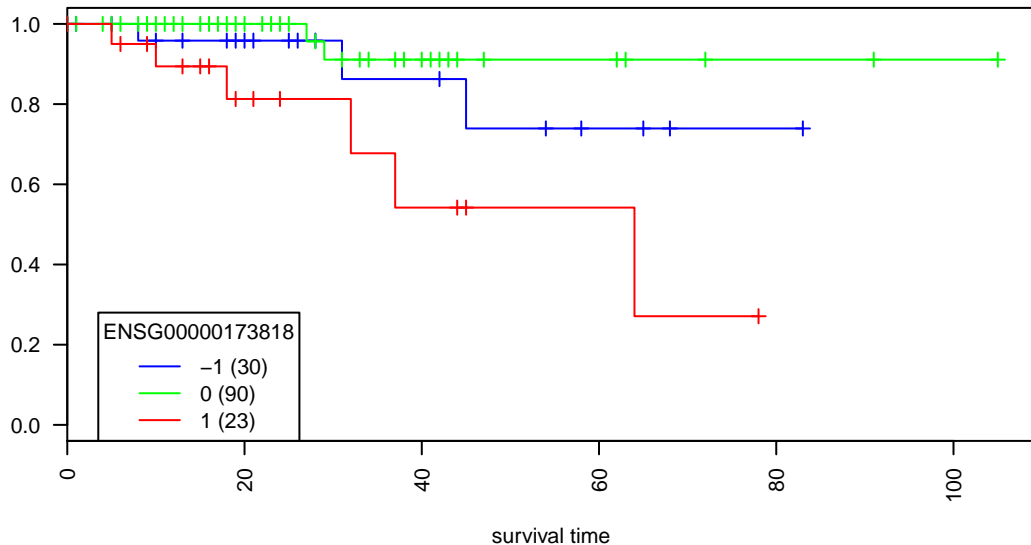

Supplement: Figure S12 — Kaplan-Meier survival plot comparing TCGA patients with overexpression (denoted 1), neutral expression (0) or underexpression (−1) of CTD-2526A2.1. Expression groups with less than 20 patients are omitted. Vertical ticks represent censoring events. The X and Y axes represent follow-up time in months and the percentage of survival, respectively. The associated log-rank p-value is 3.254192e-03. (PDF) [file pcbi.1003100.s012.pdf]

## Kaplan–Meier survival estimates

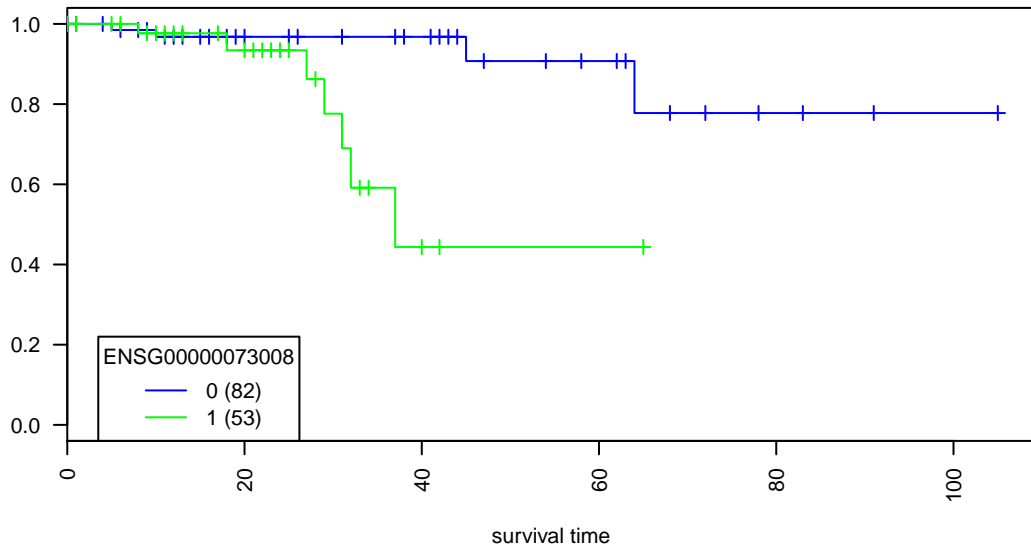

Supplement: Figure S13 — Kaplan-Meier survival plot comparing TCGA patients with overexpression (denoted 1), neutral expression (0) or underexpression (−1) of PVR. Expression groups with less than 20 patients are omitted. Vertical ticks represent censoring events. The X and Y axes represent follow-up time in months and the percentage of survival, respectively. The associated log-rank p-value is 5.386878e-03. (PDF) [file pcbi.1003100.s013.pdf]

## Kaplan-Meier survival estimates

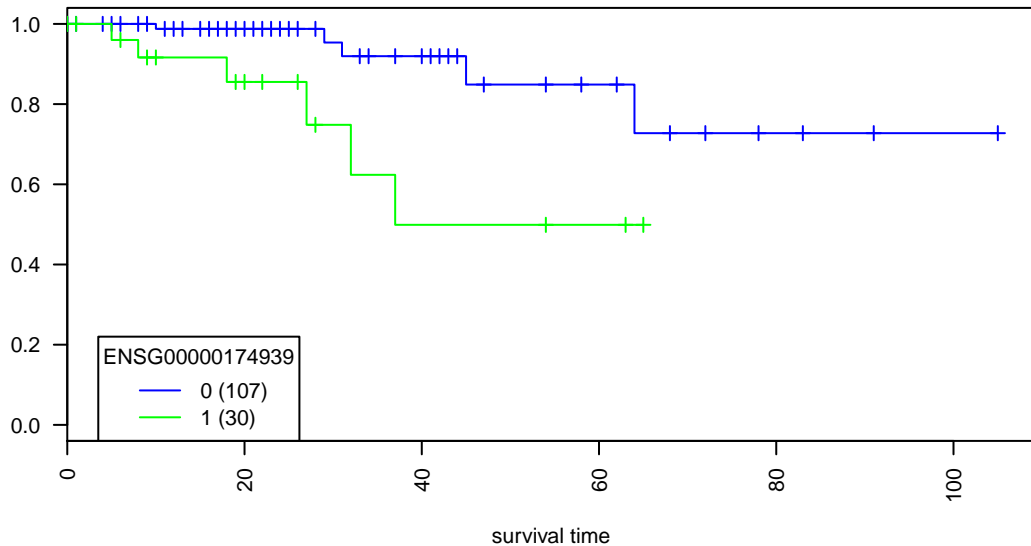

Supplement: Figure S14 — Kaplan-Meier survival plot comparing TCGA patients with overexpression (denoted 1), neutral expression (0) or underexpression (−1) of ASPHD1. Expression groups with less than 20 patients are omitted. Vertical ticks represent censoring events. The X and Y axes represent follow-up time in months and the percentage of survival, respectively. The associated log-rank p-value is 2.920611e-03. (PDF) [file pcbi.1003100.s014.pdf]

## Kaplan–Meier survival estimates

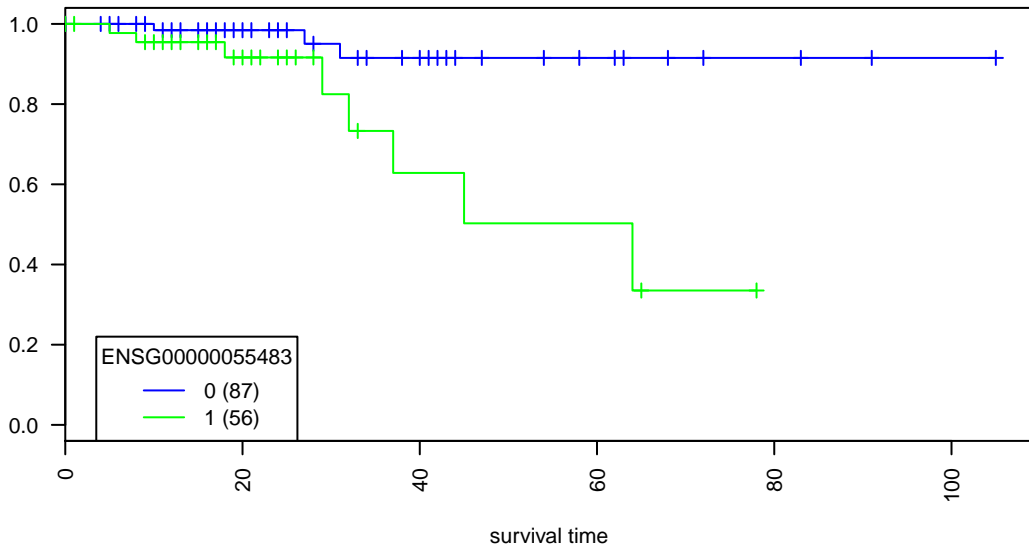

Supplement: Figure S15 — Kaplan-Meier survival plot comparing TCGA patients with overexpression (denoted 1), neutral expression (0) or underexpression (−1) of USP36. Expression groups with less than 20 patients are omitted. Vertical ticks represent censoring events. The X and Y axes represent follow-up time in months and the percentage of survival, respectively. The associated log-rank p-value is 3.151684e-03. (PDF) [file pcbi.1003100.s015.pdf]

## Kaplan-Meier survival estimates

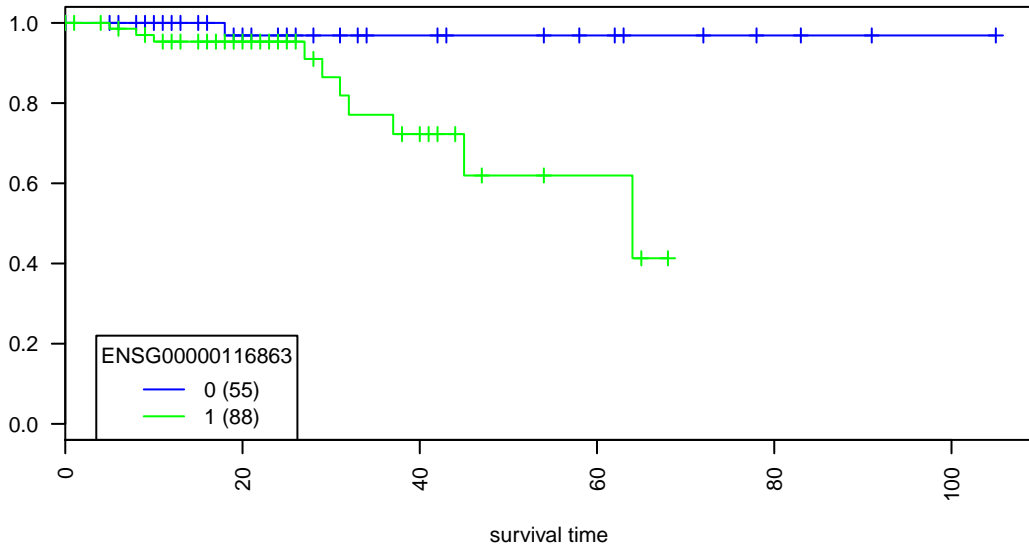

Supplement: Figure S16 — Kaplan-Meier survival plot comparing TCGA patients with overexpression (denoted 1), neutral expression (0) or underexpression (−1) of ADPRHL2. Expression groups with less than 20 patients are omitted. Vertical ticks represent censoring events. The X and Y axes represent follow-up time in months and the percentage of survival, respectively. The associated log-rank p-value is 9.95715e-03. (PDF) [file pcbi.1003100.s016.pdf]

## Kaplan–Meier survival estimates

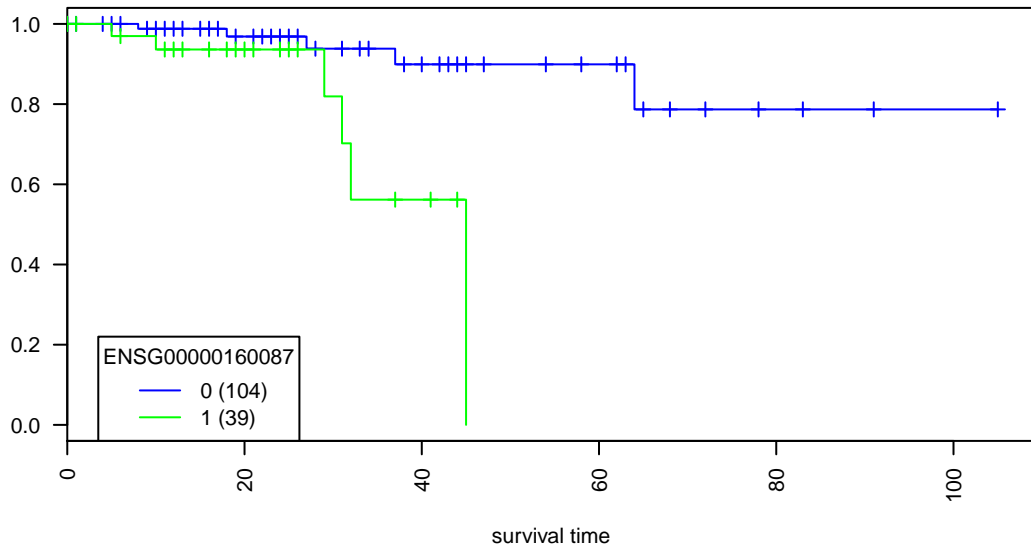

Supplement: Figure S17 — Kaplan-Meier survival plot comparing TCGA patients with overexpression (denoted 1), neutral expression (0) or underexpression (−1) of UBE2J2. Expression groups with less than 20 patients are omitted. Vertical ticks represent censoring events. The X and Y axes represent follow-up time in months and the percentage of survival, respectively. The associated log-rank p-value is 1.76163e-03. (PDF) [file pcbi.1003100.s017.pdf]

## Kaplan-Meier survival estimates

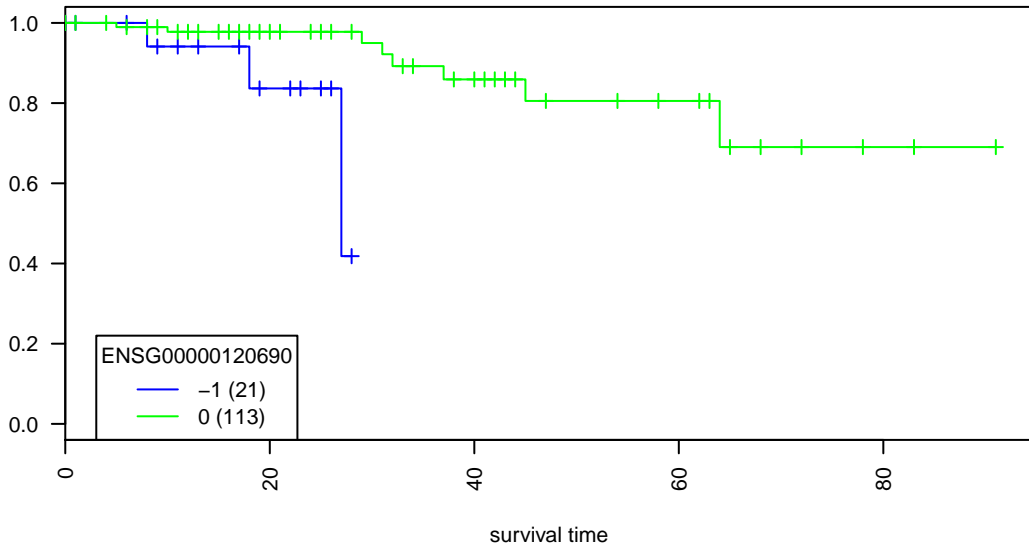

Supplement: Figure S18 — Kaplan-Meier survival plot comparing TCGA patients with overexpression (denoted 1), neutral expression (0) or underexpression (−1) of ELF1. Expression groups with less than 20 patients are omitted. Vertical ticks represent censoring events. The X and Y axes represent follow-up time in months and the percentage of survival, respectively. The associated log-rank p-value is 1.453872e-03. (PDF) [file pcbi.1003100.s018.pdf]

## Kaplan-Meier survival estimates

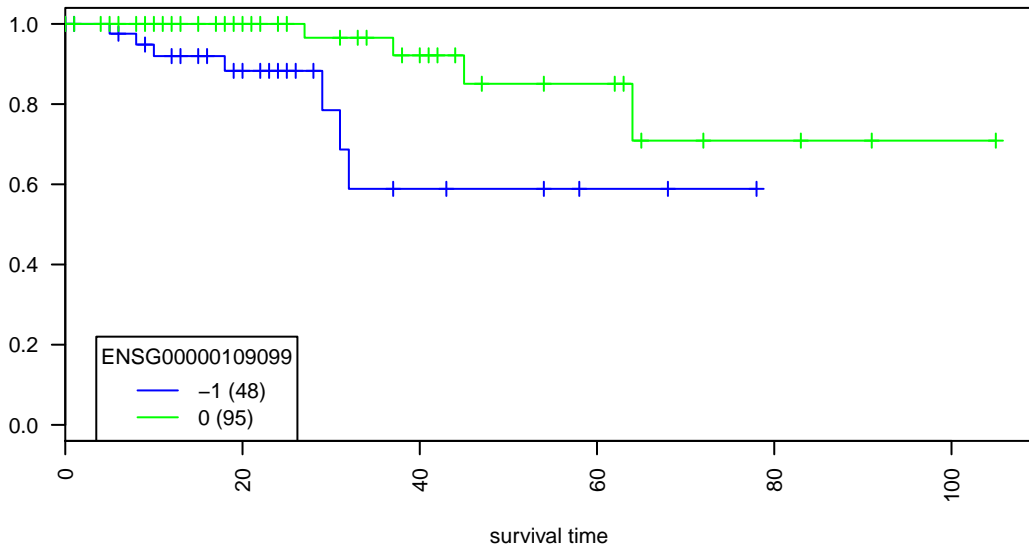

Supplement: Figure S19 — Kaplan-Meier survival plot comparing TCGA patients with overexpression (denoted 1), neutral expression (0) or underexpression (−1) of PMP22. Expression groups with less than 20 patients are omitted. Vertical ticks represent censoring events. The X and Y axes represent follow-up time in months and the percentage of survival, respectively. The associated log-rank p-value is 6.4615e-03. (PDF) [file pcbi.1003100.s019.pdf]

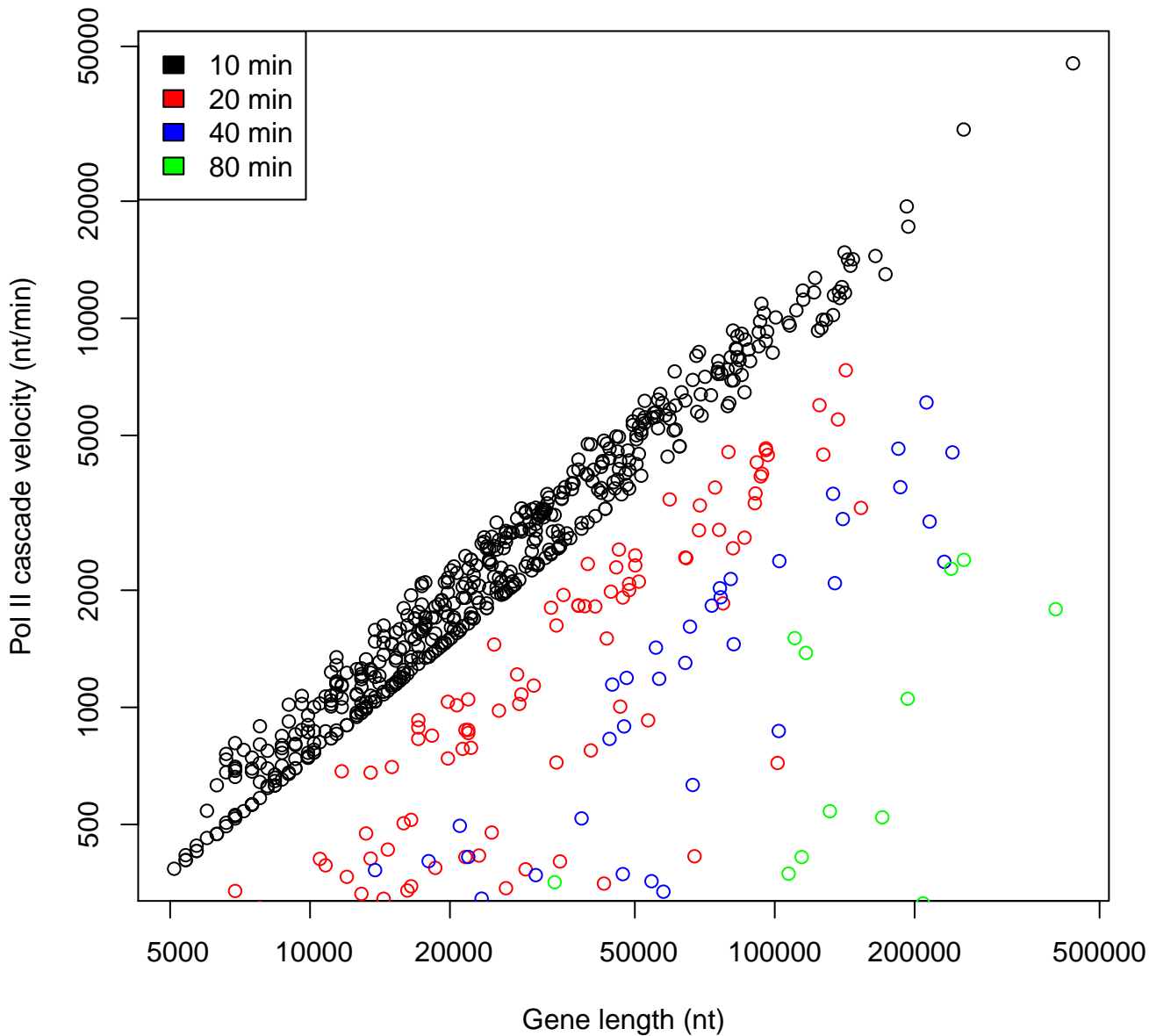

Supplement: Figure S20 — PolII elongation speed lower bound estimates compared to gene lengths. The gene set includes induced and repressed estradiol early response genes. Genes are colored accoring to the time point by which the leading or lagging end reaches the 3′ end; 80 minute time point includes genes for which the transition remains incomplete. For gene length, transcribed region lengths are used when this estimate is available; otherwise, lengths obtained from Ensembl are used. (PDF) [file pcbi.1003100.s020.pdf]

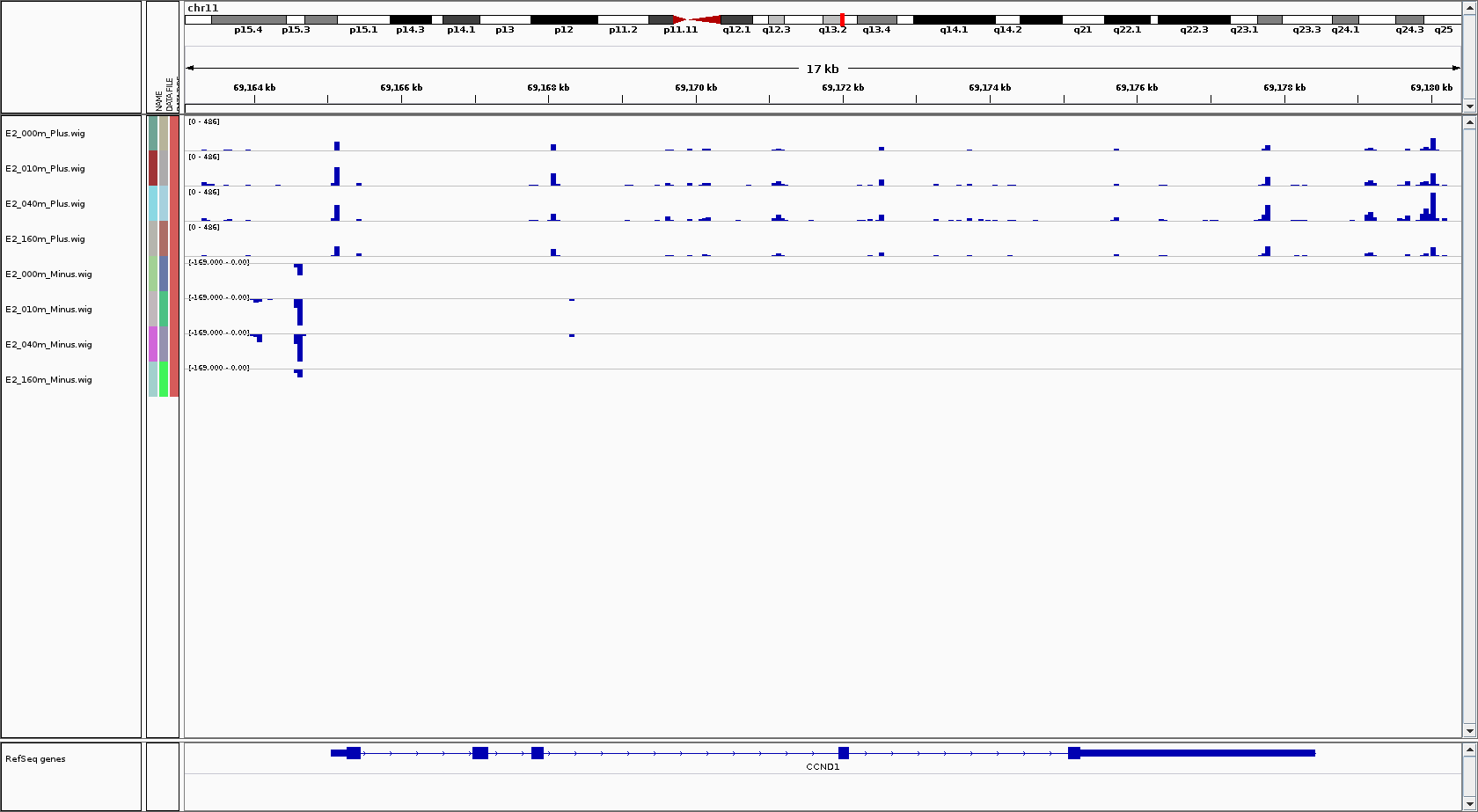

Supplement: Figure S21 — Short read counts of CCND1 in the GRO-seq experiments of [24], based on the Wiggle files in that publication. The screenshot is taken using IGV [38]. (PNG) [file pcbi.1003100.s021.png]

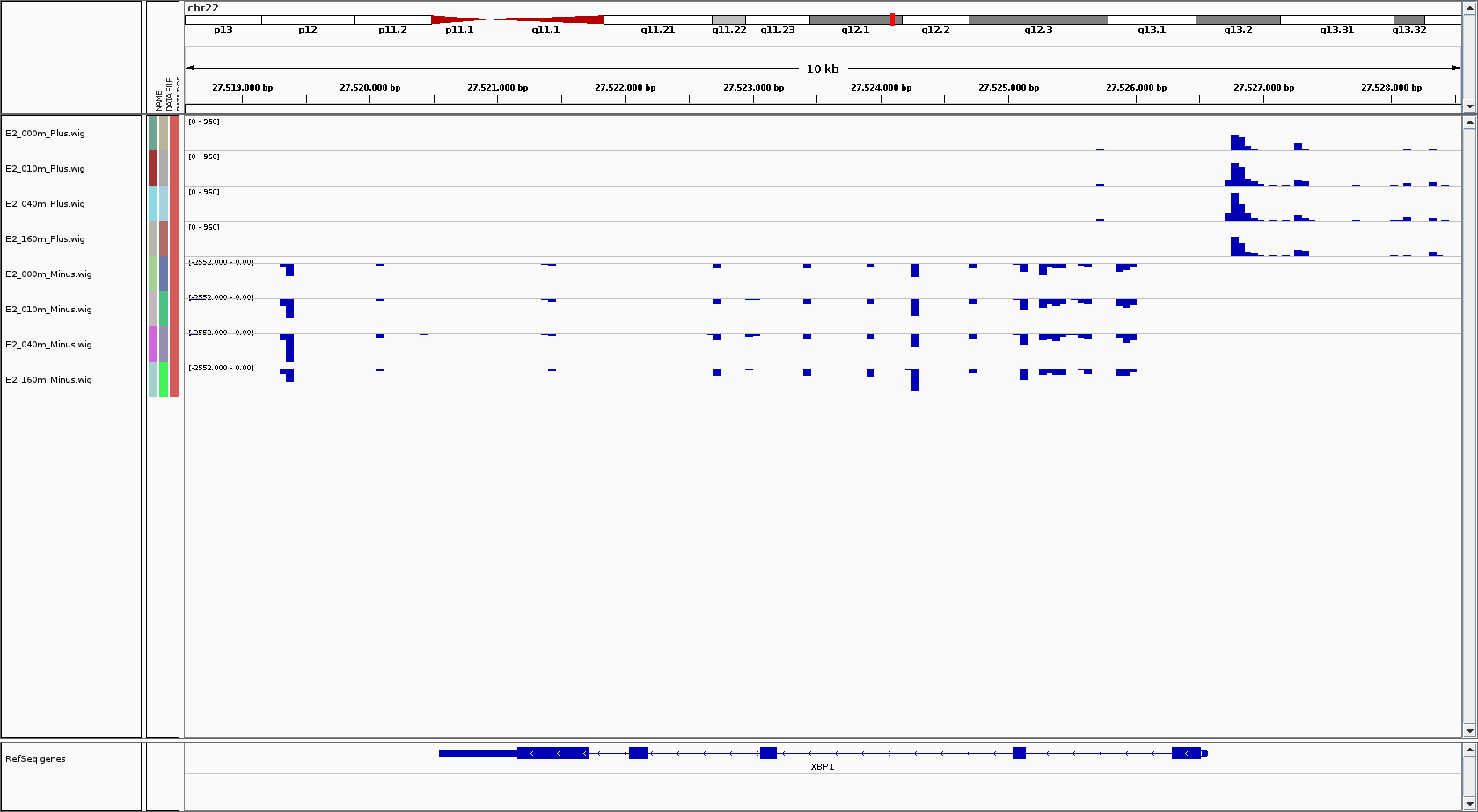

Supplement: Figure S22 — Short read counts of XBP1 in the GRO-seq experiments of [24], based on the Wiggle files in that publication. The screenshot is taken using IGV [38]. (PNG) [file pcbi.1003100.s022.png]

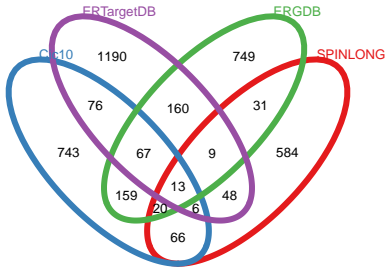

Supplement: Figure S23 — Venn diagrams for ER responsive gene set intersections. Each number denotes the number of genes in the intersection. Data for the three external gene sets are from ERGDB [26], ERTargetDB [27] and Cicatiello et al. [28] (denoted Cic10; blue). (PDF) [file pcbi.1003100.s023.pdf]

## Kaplan–Meier survival estimates

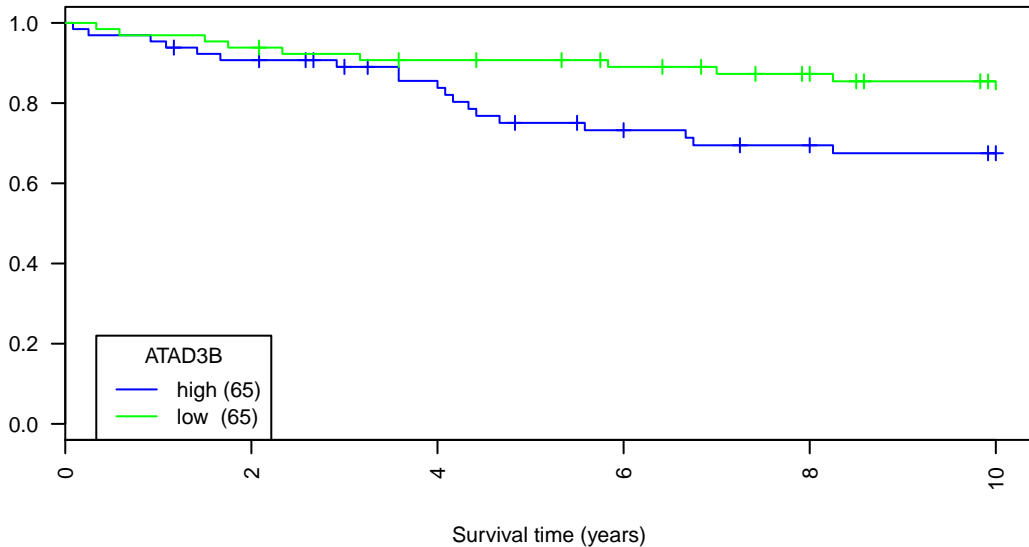

Supplement: Figure S24 — Kaplan-Meier survival plot comparing patients from [30] having above or below median expression of ATAD3B. Vertical ticks represent censoring events. Log-rank probability measure for the equality of these curves is 3.618236e-02. (PDF) [file pcbi.1003100.s024.pdf]

## Kaplan–Meier survival estimates

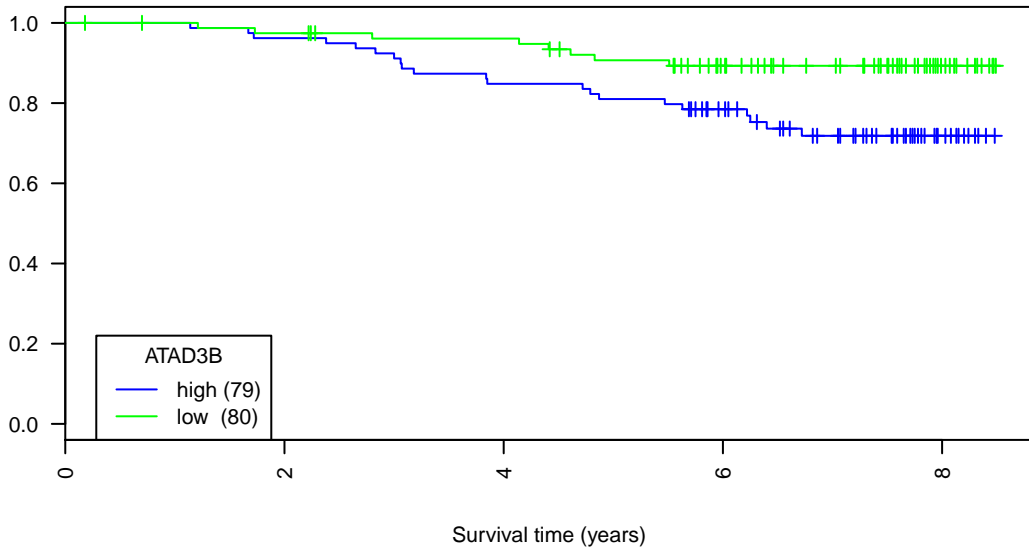

Supplement: Figure S25 — Kaplan-Meier survival plot comparing patients from [31] having above or below median expression of ATAD3B. Vertical ticks represent censoring events. Log-rank probability measure for the equality of these curves is 1.418654e-02. (PDF) [file pcbi.1003100.s025.pdf]
