# Supplementary material for: Integrative Analysis of Deep Sequencing Data Identifies Estrogen Receptor Early Response Genes and Links ATAD3B to Poor Survival in Breast Cancer
Source: PLoS Comput Biol. 2013 Jun 20;9(6):e1003100. doi: 10.1371/journal.pcbi.1003100 (PMC3688481; doi:10.1371/journal.pcbi.1003100)
Supplement: Table S1 — Survival-associated genes in the TCGA cohort predicted to respond to estradiol stimulus by SPINLONG. (PDF) [file pcbi.1003100.s026.pdf]

Gene	Survival p-value	ER binding site
ATAD3B	5.1×10^{-4}	no
BAG5	5.9×10^{-4}	yes
PPA2	1.3×10^{-3}	no
ELF1	1.5×10^{-3}	yes
UBE2J2	1.8×10^{-3}	yes
GSTM4	2.3×10^{-3}	no
ASPHD1	2.9×10^{-3}	no
VAPB	3.0×10^{-3}	no
USP36	3.2×10^{-3}	no
CTD-2526A2.1	3.3×10^{-3}	yes
SLC37A4	3.8×10^{-3}	no
CBX8	4.2×10^{-3}	yes
GPR157	4.8×10^{-3}	yes
PVR	5.4×10^{-3}	no
PMP22	6.5×10^{-3}	yes
EIF3B	7.6×10^{-3}	yes
ZNF275	8.4×10^{-3}	no
C6orf141	8.6×10^{-3}	yes
ADPRHL2	1.0×10^{-2}	no
