# Supplementary material for: Integrative Analysis of Deep Sequencing Data Identifies Estrogen Receptor Early Response Genes and Links ATAD3B to Poor Survival in Breast Cancer
Source: PLoS Comput Biol. 2013 Jun 20;9(6):e1003100. doi: 10.1371/journal.pcbi.1003100 (PMC3688481; doi:10.1371/journal.pcbi.1003100)
Supplement: Table S2 — Gene Ontology enrichment for induced genes with an binding site. (PDF) [file pcbi.1003100.s027.pdf]

GOID	Frequency	Proportion	PValueCorrected	Priori	Ontology	Description
GO:0005622	210	0.854	0.031	0.737	CC	intracellular
GO:0044424	203	0.825	0.05	0.714	CC	intracellular part
GO:0006984	9	0.038	0.05	0.007	BP	ER-nucleus signaling pathway
GO:0005737	161	0.654	0.061	0.531	CC	cytoplasm
