# Supplementary material for: Integrative Analysis of Deep Sequencing Data Identifies Estrogen Receptor Early Response Genes and Links ATAD3B to Poor Survival in Breast Cancer
Source: PLoS Comput Biol. 2013 Jun 20;9(6):e1003100. doi: 10.1371/journal.pcbi.1003100 (PMC3688481; doi:10.1371/journal.pcbi.1003100)
Supplement: Table S3 — Gene Ontology enrichment for induced genes without an binding site. (PDF) [file pcbi.1003100.s028.pdf]

GOID	Frequency	Proportion	PValueCorrected	Priori	Ontology	Description
GO:0005634	229	0.534	0	0.352	CC	nucleus
GO:0010467	187	0.469	0	0.292	BP	gene expression
GO:0090304	187	0.469	0	0.296	BP	nucleic acid metabolic process
GO:0003723	62	0.147	0	0.052	MF	RNA binding
GO:0070013	122	0.284	0	0.151	CC	intracellular organelle lumen
GO:0016070	167	0.419	0	0.259	BP	RNA metabolic process
GO:0031981	102	0.238	0	0.123	CC	nuclear lumen
GO:0016071	47	0.118	0	0.041	BP	mRNA metabolic process
GO:0034645	166	0.416	0	0.274	BP	cellular macromolecule biosynthetic process
GO:0030529	43	0.1	0	0.035	CC	ribonucleoprotein complex
GO:0005654	77	0.179	0	0.087	CC	nucleoplasm
GO:0044451	46	0.107	0	0.048	CC	nucleoplasm part
GO:0051329	29	0.073	0	0.026	BP	interphase of mitotic cell cycle
GO:0032774	127	0.318	0	0.217	BP	RNA biosynthetic process
GO:0006412	32	0.08	0	0.031	BP	translation
GO:0000082	19	0.048	0.001	0.014	BP	G1/S transition of mitotic cell cycle
GO:0006396	38	0.095	0.001	0.044	BP	RNA processing
GO:0006413	16	0.04	0.002	0.011	BP	translational initiation
GO:0006351	120	0.301	0.002	0.211	BP	transcription, DNA-dependent
GO:0006974	35	0.088	0.002	0.04	BP	response to DNA damage stimulus
GO:0000084	14	0.035	0.003	0.009	BP	S phase of mitotic cell cycle
GO:0036094	98	0.232	0.006	0.159	MF	small molecule binding
GO:0000216	10	0.025	0.008	0.005	BP	M/G1 transition of mitotic cell cycle
GO:0006397	25	0.063	0.008	0.026	BP	mRNA processing
GO:0051252	115	0.288	0.008	0.207	BP	regulation of RNA metabolic process
GO:0005840	17	0.04	0.009	0.013	CC	ribosome
GO:0000166	91	0.216	0.01	0.147	MF	nucleotide binding
GO:0000502	9	0.021	0.01	0.004	CC	proteasome complex
GO:0016851	4	0.009	0.012	0	MF	magnesium chelatase activity
GO:0015979	4	0.01	0.012	0.001	BP	photosynthesis
GO:0015995	4	0.01	0.012	0.001	BP	chlorophyll biosynthetic process
GO:0010468	124	0.311	0.013	0.231	BP	regulation of gene expression
GO:0042555	4	0.009	0.015	0.001	CC	MCM complex
GO:0003735	14	0.033	0.015	0.01	MF	structural constituent of ribosome
GO:0003743	8	0.019	0.016	0.004	MF	translation initiation factor activity
GO:0031571	9	0.023	0.018	0.005	BP	mitotic cell cycle G1/S transition DNA damage checkpoint
GO:0004386	14	0.033	0.018	0.011	MF	helicase activity
GO:0044391	12	0.028	0.022	0.008	CC	ribosomal subunit
GO:2000112	116	0.291	0.022	0.217	BP	regulation of cellular macromolecule biosynthetic process
GO:0006271	6	0.015	0.024	0.002	BP	DNA strand elongation involved in DNA replication
GO:0008380	20	0.05	0.029	0.021	BP	RNA splicing
GO:0030330	11	0.028	0.03	0.008	BP	DNA damage response, signal transduction by p53 class mediator
GO:0006355	108	0.271	0.032	0.201	BP	regulation of transcription, DNA-dependent
GO:0005739	60	0.14	0.033	0.091	CC	mitochondrion
GO:0006977	8	0.02	0.033	0.004	BP	DNA damage response, signal transduction by p53 class mediator resulting in cell cycle arrest
GO:0016604	17	0.04	0.033	0.016	CC	nuclear body
GO:0000932	6	0.014	0.036	0.002	CC	cytoplasmic mRNA processing body
GO:0035308	3	0.008	0.037	0	BP	negative regulation of protein dephosphorylation
GO:0005671	4	0.009	0.037	0.001	CC	Ada2/Gcn5/Ada3 transcription activator complex
GO:0015934	8	0.019	0.046	0.004	CC	large ribosomal subunit
GO:0003677	85	0.201	0.05	0.145	MF	DNA binding
GO:0006270	5	0.013	0.054	0.002	BP	DNA-dependent DNA replication initiation
GO:0016363	8	0.019	0.055	0.005	CC	nuclear matrix
GO:0031124	8	0.02	0.055	0.005	BP	mRNA 3'-end processing
GO:0016783	3	0.007	0.061	0	MF	sulfurtransferase activity
GO:0044265	32	0.08	0.063	0.045	BP	cellular macromolecule catabolic process
GO:0051151	3	0.008	0.063	0	BP	negative regulation of smooth muscle cell differentiation
GO:0006366	54	0.135	0.063	0.089	BP	transcription from RNA polymerase II promoter
GO:0006281	22	0.055	0.063	0.027	BP	DNA repair
GO:0005730	28	0.065	0.064	0.035	CC	nucleolus
GO:0019058	15	0.038	0.066	0.015	BP	viral infectious cycle
GO:0000375	14	0.035	0.07	0.014	BP	RNA splicing, via transesterification reactions
GO:0016442	3	0.007	0.071	0	CC	RNA-induced silencing complex
GO:0004529	3	0.007	0.071	0	MF	exodeoxyribonuclease activity
GO:0016895	3	0.007	0.071	0	MF	exodeoxyribonuclease activity, producing 5'-phosphomonoesters
GO:0006268	3	0.008	0.074	0.001	BP	DNA unwinding involved in replication
GO:0000956	12	0.03	0.074	0.011	BP	nuclear-transcribed mRNA catabolic process
GO:0051443	8	0.02	0.075	0.005	BP	positive regulation of ubiquitin-protein ligase activity
GO:0005838	3	0.007	0.087	0.001	CC	proteasome regulatory particle
GO:0051436	7	0.018	0.091	0.004	BP	negative regulation of ubiquitin-protein ligase activity involved in mitotic cell cycle
GO:0016605	7	0.016	0.096	0.004	CC	PML body
